# ZetaSuite: computational analysis of two-dimensional high-throughput data from multi-target screens and single-cell transcriptomics

**DOI:** 10.1186/s13059-022-02729-4

**Published:** 2022-07-25

**Authors:** Yajing Hao, Shuyang Zhang, Changwei Shao, Junhui Li, Guofeng Zhao, Dong-Er Zhang, Xiang-Dong Fu

**Affiliations:** 1 Department of Cellular and Molecular Medicine, Institute of Genomic Medicine, University of California San Diego, La Jolla, CA 92093 USA; 2 29 Rosedale Ave, MA 01545 Shrewsbury, USA; 3 Howard Hughes Medical Institute, Department of Medicine, University of California, San Diego, La Jolla, CA 92093 USA; 4 Moores Cancer Center, Department of Biological Sciences, Department of Pathology, University of California, San Diego, La Jolla, CA 92093 USA

**Keywords:** Zeta statistics, two-dimensional RNAi screening, Single-cell RNA-seq, Cancer dependency, Cancer checkpoint

## Abstract

Two-dimensional high-throughput data have become increasingly common in functional genomics studies, which raises new challenges in data analysis. Here, we introduce a new statistic called Zeta, initially developed to identify global splicing regulators from a two-dimensional RNAi screen, a high-throughput screen coupled with high-throughput functional readouts, and ZetaSuite, a software package to facilitate general application of the Zeta statistics. We compare our approach with existing methods using multiple benchmarked datasets and then demonstrate the broad utility of ZetaSuite in processing public data from large-scale cancer dependency screens and single-cell transcriptomics studies to elucidate novel biological insights.

**Supplementary Information:**

The online version contains supplementary material available at 10.1186/s13059-022-02729-4.

## Background

Genome-wide screen by RNA interference (with siRNA or shRNA) [1–3] or CRISPR/Cas (with sgRNA) [4–6] has become a powerful tool for functional genomics studies. Most studies monitor a single functional readout in one-dimensional high-throughput screens or a few functional consequences in so-called high-content screens [1, 7]. By leveraging the power of deep sequencing, it has become feasible to simultaneously quantify the expression of a gene signature consisting of hundreds or even thousands of genes in two-dimensional high-throughput screens [8, 9]. shRNA or sgRNA libraries have also been used to treat hundreds of cell lines to deduce genes whose depletion compromise cancer cell growth, referred to as cancer dependencies [10–13], which presents a type of two-dimensional screens. Single-cell transcriptomics and multi-omics studies are also examples of two-dimensional and even multi-dimensional high-throughput data for integrated analysis of regulated gene expression in individual cells [14].

The increasing power of next-generation sequencing has thus made it feasible and cost-effective to generate multi-dimensional high-throughput data to gain deeper understanding of regulatory biology. The advance in high-throughput technologies is also frequently accompanied by the demand for developing new analytical tools to process data of increasing complexity. For one-dimensional high-throughput screens, *t*-test, Z-statistics or Robust Z-statistics, or strictly standardized mean difference (SSMD) or Robust SSMD [15] have been typically employed to identify screen hits, depending on the availability of replicates and built-in positive and/or negative controls [16]. However, as demonstrated in this study, these simple statistical approaches are no longer suitable for analyzing two-dimensional high-throughput data.

Single-cell transcriptomics has become a powerful tool to study regulated gene expression in individual cells [17, 18]. Due to highly stochastic sampling in single cells during library construction, it is critical to identify high-quality cells for subsequent clustering and trajectory analyses [19]. Three methods implemented in Seurat, CellRanger, and EmptyDrops have been commonly used for quality control (QC) purpose: Seurat [20] allows users to choose arbitrary thresholds to remove low-quality cells based on nFeature (the number of expressed genes detected), nCount (total reads), or %mt (percentage of mitochondrial transcripts). Based on nCount alone CellRanger sets the inflection point as the threshold in a knee-plot, which tends to miss smaller cells with relatively lower nCount values. EmptyDrops [21] is designed to “rescue” some of those missed cell populations by simulating the level of ambient RNA (those from lysed cells, not from a specifically barcoded cell), but at the expense of contamination with other low-quality cells. Notably, each of these approaches still relies on a single parameter, rather than integrates multiple parameters, for making a cutoff in analyzing single-cell transcriptomics data.

In this study, we recognize the challenges in treating two-dimensional high-throughput data with existing methods, which has motivated us to develop a new statistics called Zeta by taking two critical QC metrics into consideration. We also establish a corresponding software package ZetaSuite to facilitate its application (https://github.com/YajingHao/ZetaSuite). Using our own RNAi screen data, we use ZetaSuite to minimize noise accumulation in comparison with multiple existing methods, aid in hit selection based on the newly proposed Screen Strength, and pinpoint likely off-targets. We also illustrate the robustness of ZetaSuite in processing two sets of large-scale cancer dependency datasets, revealing new cancer dependencies and uncovering novel cancer checkpoints. Finally, we demonstrate the advantage of ZetaSuite in identifying high quality single cells while excluding empty and broken droplets in single cell transcriptomics analysis. Collectively, these applications showcase the broad utility of ZetaSuite in processing diverse two-dimensional high-throughput data to reveal novel biological insights.

## Results

### Overview of the ZetaSuite workflow

**Fig. 1:**
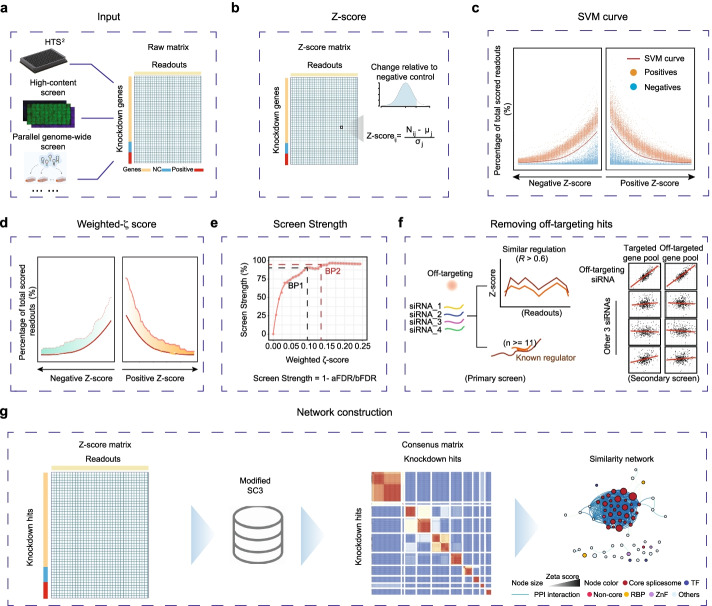
Overview of the ZetaSuite workflow. **a** Two-dimensional screens include high-throughput screen by high through sequencing (HTS^2^), high-content screen, parallel genome-wide screens, etc. ZetaSuite uses the raw matrix as input to calculate *ζ* score. **b–g** Key steps in the ZetaSuite method from generating initial *ζ* scores (**b**) to deducing hits by using negative and positive controls to derive a support vector machine (SVM) learning curve (**c**) to calculating weighted *ζ* scores (**d**) to determining the Screen Strength (**e**) to filtering out off-targets (**f**). The resulting data are used to construct regulatory gene networks based on functional similarities (**g**)

ZetaSuite is a computational framework initially developed to process the data from a siRNA screen for global splicing regulators. In this screen, we interrogated ~400 endogenous alternative splicing (AS) events by using an oligo ligation-based strategy and quantified their responses to 18,480 pools of siRNAs against annotated protein-coding genes in the human genome (Additional file 1: Fig. S1a). We next performed deep sequencing on pools of bar-coded samples from individually treated wells in 384-well plates to generate digital information on individual mRNA isoforms. By comparing with internal non-specific siRNA-treated samples, we quantified induced exon inclusion or skipping for each AS event (similar to up- and down-regulated genes from RNA-seq experiments). The resultant data matrix resembled those produced by high-content screens, parallel genome-wide screens, or any screens that monitor multiple functional outcomes (Fig. 1a), emphasizing the broad applicability of ZetaSuite (outlined in Additional file 1: Fig. S1b) for processing two-dimensional high-throughput data, even though we presently focus on using our own RNAi screen data to develop the Zeta statistics underlying ZetaSuite (see below).

After a series of standard data pre-processing and QC steps, ZetaSuite generates a *Z*-score for each AS event against each targeting RNA in the data matrix (Fig. 1b) and then computes the number of hits at each *Z*-score cutoff from low to high and in both directions to separately quantify induced exon skipping (Fig. 1c, left) or inclusion (Fig. 1c, right) events. This enables classification of functional data in both directions to identify and characterize global splicing activators (if mostly causing exon skipping upon knockdown) or repressors (if mostly inducing exon inclusion events upon knockdown) or both. The same strategy can be used to characterize positive and negative regulators in other biological contexts.

In applications where internal positive controls are well separated from negative controls, as showcased with our RNAi screen dataset, ZetaSuite calculates an SVM learning curve to maximally separate positives from negatives. Any siRNA that generates a line (a string of data points in the plot) above the SVM line would be considered a potential hit and the area between the two lines could be used to quantify the strength of the hit, thus enabling rank-order individual hits (Fig. 1d). We name this statistics as *Z*-based estimate of targets or Zeta (*ζ*). Even without positive controls in certain applications, it is still possible to calculate the area between each data line and the x-axis to generate a *ζ* score for a given hit.

As with all screens, a threshold needs to set for hit calling. To this end, we utilize a large set of non-expressed genes in a given cell type (HeLa cells in our screen) as internal negative controls and determines the number of hits above a given *ζ* score to plot against the number of non-expressed genes mistakenly identified as hits (which may result from non-specific perturbations or off-target effects). We call this a Screen Strength (SS) plot and select a balance point as the threshold where a further increase in *ζ* score no longer significantly improves the value of the SS (Fig. 1e). Last, but not least, ZetaSuite also takes full advantage of two-dimensional high-throughput data to calculate similarities in global responses through pairwise comparisons, which could be leveraged to deduce off-target effects based on the results from the secondary screen (Fig. 1f), and, more importantly, to construct gene networks for functional analysis of screen hits (Fig. 1g). ZetaSuite thus provides a comprehensive package for analyzing two-dimensional high-throughput data. Below, we describe how the Zeta statistics is progressively developed in addressing challenges in processing two-dimensional high-throughput data in comparison with multiple existing methods and demonstrate the utility of ZetaSuite in analyzing representative two-dimensional high-throughput data to reveal novel biological insights.

### Increasing readout number leads to diminishing screen specificity with traditional methods

**Fig. 2:**
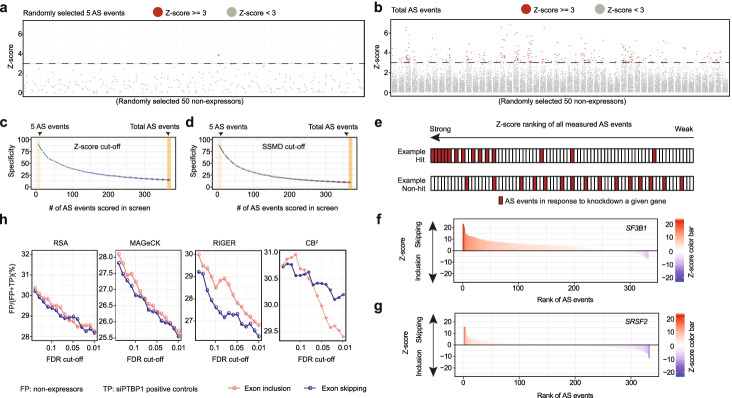
Increasing readout number leads to diminishing screen specificity with common statistical approaches. **a**, **b** The distribution of Z-scores based on 5 randomly selected alternative splicing (AS) events monitored in our screen (**a**) or all AS events measured (**b**) in response to siRNAs against 50 randomly selected non-expressed genes. The AS event was marked as red if the *Z*-score is >=3. **c**, **d** The Specificity based on common cutoffs (**c**, *Z*-score>=3) or SSMD value (**d**, SSMD value>=2) when different numbers of AS events were monitored. The specificity (defined by 1 minus the number of non-expressors scored as hits over the total number of non-expressors) is the mean value of 50 replicates under each condition. **e** Illustration of the principal theory to determine hits based on RSA, MAGeCK, and RIGER. Induced changes in AS are first ranked and the effects of knocking down a given gene on individual AS events are displayed as red bars. A hit would show enriched AS events in one direction (top) while a non-hit would display a relatively random distribution (bottom). **f**, **g** The distribution of induced AS events (based on *Z*-scores of induced exon skipping from left to right at top or induced exon inclusion from right to left at bottom) in response to knockdown *SF3B1* (**f**) or *SRSF2* (**g**). **h** The false discovery rate (FDR=FP/(FP+TP)) at different cutoffs with different methods. The FDRs at *x*-axis were calculated by different software (RSA, RIGER, MAGeCK, and CB^2^). The FDRs at y-axis were deduced based on the non-expressors and built-in positive controls (siPTBP1). False positive (FP): non-expressors; true positive (TP): siPTBP1-treated samples

Z-statistics or SSMD has been typically used to identify hits from one-dimensional high-throughput screen data. SSMD has advantages if a screen includes multiple replicates for each targeting RNA [16]. When the number of screen readouts increases, however, various random outliers become accumulated, which has the potential to severely compromise the screen specificity. For instance, we scored ~400 AS events against each siRNA with 368 events passing data QC requirements (see Methods). If any of these readouts meets a chosen cutoff, the probability of experimental noise and/or off-target effects would be aggregated in proportion to the number of readouts scored. To demonstrate this, we chose a stringent cutoff of *Z*-score>=3 [22] to identify hits from our splicing screen data and used siRNAs that target non-expressed genes as true negatives to estimate the screen specificity. Randomly selecting 50 siRNAs against non-expressed genes based on 5 randomly selected AS events, we identified 1 hit out of 50 true negative siRNAs (Fig. 2a). When all 368 AS events scored on our screen were taken into consideration, the majority of those true negative siRNAs became hits (Fig. 2b).

This alarming high false-positive rate became further evident when all RNA-seq identified non-expressed genes were included in the analysis (Additional file 1: Fig. S2a-b). By selecting an increasing number of AS events as readouts to determine the screen specificity, we found that the screen specificity was progressively decreased (Fig. 2c), and we obtained the same result by performing a similar analysis based on SSMD (Fig. 2d). These data illustrate that the most popular statistical approaches for analyzing one-dimensional screen data are no longer suitable for processing two-dimensional high-throughput data. Even after using the multiple testing correction methods (such as FDR and Bonferroni correction, as well as coupling with the Gumbel distribution, see Methods), the error rate is still very high.

Next, we wondered whether we might adapt the concept from some more sophisticated methods to analyze two-dimensional high-throughput data. For example, RSA [23], RIGER [24], MAGeCK [25], and CB^2^ [26] were each designed to determine the impact of a given gene on a functional readout (e.g., cell proliferation) by testing multiple targeting RNAs against each gene and then aggregating the data to reflect the overall contribution of such gene to the functional consequence. A typical data aggregation strategy is analogous to Gene Set Enrichment Analysis (GSEA) [27], which is to first rank order all targeting RNAs against all targeted genes tested based on the functional impact measured in the screen (e.g., the impact on cell proliferation from high on left to low on right) and then score hits if multiple targeting RNAs are relatively enriched toward left (Fig. 2e, top raw) whereas a non-hit lacks any enrichment (Fig. 2e, bottom raw).

Here, by replacing individual targeting RNAs with individual AS events, we took a similar strategy to evaluate the overall contribution of a given gene to global splicing control. Using two well-known splicing regulators as benchmarks and separately rank ordering their impact on exon skipping (left to right) or inclusion (right to left), we found that knockdown of the core spliceosome component SF3B1 mainly caused exon skipping (Fig. 2f and Additional file 1: Fig. S2c), whereas depletion of a representative SR protein SRSF2 induced both exon inclusion and skipping in about equal frequency (Fig. 2g and Additional file 1: Fig. S2d). These data are well in line with the existing literature [28, 29]. Extending this analysis genome-wide, we identified thousands of genes as putative splicing regulators by using different aggregation strategies associated with RSA, RIGER, MAGeCK, or CB^2^ (Additional file 1: Fig. S2e). We next took advantage of 5006 siRNAs against non-expressed genes as internal negative controls and 299 technical repeats with an siRNA against a well-known splicing regulator PTBP1 [30] as internal positive controls in our screen and estimated the false discovery rate (FDR=false positives divided by false positives + true positives). We observed an alarmingly high error rate with each of these methods even at the most stringent FDR cutoff (Fig. 2h). Collectively, these analyses present a compelling paradigm for the need to develop new statistics to fully explore the power of two-dimensional high-throughput data.

### Zeta: Z-based estimation of global splicing regulators

**Fig. 3:**
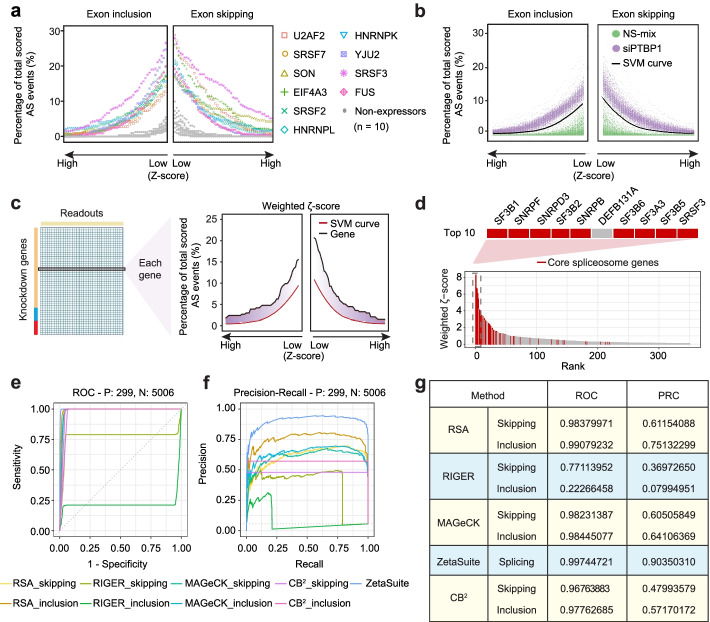
The *ζ* and comparison with several key existing statistical approaches. **a** At each *Z*-score bin over a full Z-score range, the level of hits (expressed as the percentage of induced AS events over the total number of AS events monitored) is plotted with 10 representative splicing regulators (individually colored) compared to 10 non-expressors (grey). Left and right separately plot induced exon inclusion and skipping events. **b** At each *Z*-score bin over a full *Z*-score range, the level of hits in response to siPTBP1 (purple) or negative controls (NS-mix, green). An optimal SVM curve (black) is derived to maximally distinguish between true positives (siPTBP1) and true-negatives (NS-mix). **c** Calculation of a weighted *ζ*-score based on the area between the specific *Z*-score line of a gene (black) and the SVM curve (red). At each *Z*-score bin, the area is calculated by multiplying the *Z*-score, thus giving increasingly weights (purple) to hits at higher *Z*-scores. **d** The distribution of weighted *ζ*-score for annotated core spliceosome components among top 350 high-ranking genes. The top 10 high-ranking genes are enlarged (top). Only *DEFB131A* doesn’t belong to core spliceosome, which was later determined to result from off-targeting to *SF3B1* (see Additional file 1: Fig. S4d). **e**, **f** The ROC (**e**) and PRC (**f**) curves are deduced using different software. Weighted *ζ*-score in two directions calculated by ZetaSuite are combined in this analysis to reflect the overall functional consequence. This is not applicable to other software, and we thus display the data separately. **g** The summary of the areas under all deduced ROC and PRC curves using different software

It becomes quite evident from the above analyses that the accumulation of random experimental noise and off-target effects is a major problem in analyzing two-dimensional high-throughput data because the screen specificity is progressively diminished as the number of readouts increases. To begin to develop a new statistical strategy to address this problem, we first used non-expressed genes to characterize the distribution of random splicing responses from all AS events quantified on our screen. For each siRNA against a given non-expressed gene, we calculated *Z*-scores for the entire collection of the AS events scored and then displayed the number of “hits” at each *Z*-score cutoff from low to high for induced exon skipping (toward the right) or exon inclusion (toward the left). This shows the progressive decline in the number of hits in both directions as the *Z*-score value increases, and after analyzing 10 randomly selected non-expressed genes this way, we noted that all exhibit a similar distribution (Fig. 3a, grey color). In comparison, among 10 representative splicing regulators (Additional file 1: Fig. S3a), all scored a much higher number of hits at any *Z*-score cutoff (Fig. 3a, individually colored).

Interestingly, such distinct profiles between non-expressors and known splicing regulators were similarly observed with a large number of built-in negative controls (NS-mix, a pool of non-specific siRNAs) and positive controls (siPTBP1, a specific siRNA pool against *PTBP1*). This enabled us to develop an SVM curve to maximally separate positives from negatives (Fig. 3b). We define the area between a putative hit above the SVM line as a *Z*-based estimate of targets or Zeta (*ζ*). In order to favor the differences at higher *Z*-score cutoffs, we recommend the use of a weighted *ζ* score, which is calculated as follows: we first divide the full Z-score range into 100 bins, multiply the averaged Z-score value by the area at each bin, and finally aggregate values from all 100 bins (Fig. 3c, see Methods for further details). This generates a weighted-*ζ* score to define the overall impact of a putative splicing regulator.

To characterize a given splicing regulator in splicing activation and repression, we separately calculated *ζ* scores for aggregated exon inclusion or skipping events. After processing our splicing screen data with this analysis pipeline (ZetaSuite, see Additional file 1: Fig. S1b), we rank-ordered the hits according to their overall impact on AS (high to low from left to right), thus enabling quantification of each splicing regulators based on its global contribution to regulated splicing in a given cell type. Interestingly, we noted that most high-ranking hits correspond to annotated core spliceosome components (Fig. 3d). This suggests that components of the core splicing machinery also function as the most prevalent class of AS regulators in mammalian cells. In general, these genes are highly expressed in mammalian cells and their inactivation predominantly induces exon skipping (Additional file 1: Fig. S3b-c).

To compare the performance of the newly developed *ζ* statistics with other ranking approaches, such as that used in RSA, RIGER, MAGeCK, or CB^2^, we again took advantage of a large number of built-in positive and internal negative controls in our screen, which allowed us to precisely determine the numbers of true and false positives and negatives to construct receiver operating characteristic (ROC) (Fig. 3e) and precision-recall curves (PRC) (Fig. 3f). Additionally, as the *ζ* statistics is designed to deal with random error accumulation due to increasing readout numbers, we generated a set of simulated datasets based on our two-dimensional splicing screen datasets by randomly selecting readouts from our raw datasets (see Methods). The *ζ* statistics again outperformed all aforementioned methods, as shown by the calculated values of areas under PRCs or AUPRCs (Fig. S3d). Together, these comparisons demonstrate that the newly developed *ζ* statistics significantly outperformed all other ranking methods in analyzing two-dimensional high-throughput splicing screen data (Fig. 3g).

### Selecting hits based on the reflection point in Screen Strength plot

Any screen requires a cutoff to maximize positives and minimize negatives. In most one-dimensional high-throughput screens, hits are first ranked based on *Z*-score or SSMD values followed by the selection of a threshold by estimating the false positive level (FPL) and the false negative level (FNL) [31]. As *Z*-score or SSMD value increases, FPL gradually decreases while FNL progressively increase [32]. This approach can be similarly applied to *ζ*-based scoring, as illustrated with our splicing screen data using siPTBP1 in technical repeats as true positives and siRNAs against non-expressed genes as true negatives (Additional file 1: Fig. S4a). Using the balanced error level approach as recommended earlier [31], we obtained 10% for both FPL and FNL with a calculated FDR of 15.4%. However, many siRNA screens may not be able to build in sizable true positive controls and the balanced error level may be influenced by the ability to efficiently differentiate between positive and negative controls. To address this problem, RNAiCut was developed to identify an appropriate cutoff for hit selection by coupling the orthogonal PPI network information [33]. We noted that RNAiCut heavily depends on the accuracy of the established PPI networks, which is challenging in mammalian cells. Additionally, we further noted that the recommended minimum p-value selection as cutoff is not always true, especially for some specific functions that need the incorporation of multiple pathways.

**Fig. 4:**
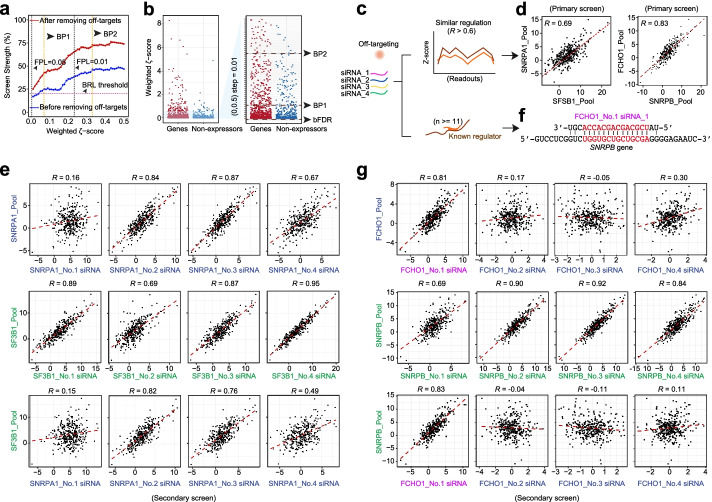
Hit selection based on Screen Strength and strategy to filter out off-target effects. **a** The comparison of the Screen Strength before (blue) and after (red) filtering out off-targets. BP: balance point. Note that the Screen Strength based on the threshold defined by the commonly used balanced error level (BRL) approach is also indicated (see Additional file 1: Fig. S4**a**). Empirical FPL lines (0.05 and 0.01) are also indicated. Those between BP1 and BP2 are candidate hits and those after BP2 are high confidence hits. **b** Weighed *ζ*-scores of expressed and non-expressed genes. A specific region is enlarged on the right for comparative purpose. bFDR: baseline FDR. BP1 and BP2 are according to those defined in **a**. **c** Strategy to filter out off-target effects based on similarity in response and sequence complementarity. **d** Comparison of AS events responsive to knockdown of *SNRPA1* and *SF3B1* or *SNRPB* and *FCHO1* in primary screen. Pearson correlation score is indicated in each case. **e** Comparison of AS events responsive to knockdown of the siRNA pool vs individual siRNAs against *SNRPA1* or *SF3B1* in the secondary screen. The third row shows the comparison between the siRNA pool against *SF3B1* and individual siRNAs against *SNRPA1*. **f** The sequence of a single siRNA targeting *FCHO1* is aligned with its potential off-target on the *SNRPB* transcript. **g** Comparison of AS events responsive to knockdown of the siRNA pool vs individual siRNAs against *FCHO1* or *SNRPB* in the secondary screen. The third row shows the comparison between the siRNA pool against *SNRPB* and individual siRNAs against *FCHO1*. Purple highlights the predicted off-targeting siRNA

Given these challenges, we introduce the concept of apparent FDR (aFDR), which is defined as the number of non-expressors identified as false positive hits among all hits scored at a given cutoff. Before the screening, we had a baseline FDR (bFDR), which corresponded to the number of non-expressors among the total number of genes targeted in the screen. By definition, bFDR represents the chance from a random draw. We next define the Screen Strength: SS=1-aFDR/bFDR, which can be used to evaluate the effectiveness a screen has achieved relative to a random draw. We applied this approach to generate the SS plot based on the splicing screen data against increasing *ζ* scores (Fig. 4a). This allowed us to calculate a balance point (BP) for hit selection where the SS remains almost little change as the stringency increases. We actually identified two such BPs with our splicing screen data, thereby defining candidate hits after BP1 and high confidence hits after BP2, the latter of which maximally eliminate true false positives derived from non-expressors (Fig. 4b).

To demonstrate the broad utility of SS, we utilized 5 public RNAi screen datasets [34–38] to select appropriate cutoffs (Fig. S4b). Interestingly, each of these high-quality screen results exhibited two apparent balance points. This is anticipated if the ranking values had the ability to differentiate between positives and negatives. To demonstrate this, we permutated the ranking values of a representative genome-wide screen (from the last dataset in Fig. S4b) five times and found that we were no longer be able to detect any balance point from the SS plot generated with the permutated dataset. We thus suggest that the SS plot is generally applicable to selecting a cutoff(s) and the presence of at least one balance point is indicative of a successful screening dataset.

### Strategy to remove off-target effects from two-dimensional high-throughput RNAi screens

Off-target effects have been a major problem in genome-wide screens. Recent strategies to filter out off-targeting RNAs are to increase the number of targeting RNAs against each gene and eliminate those that show divergent effects from the consensus generated by multiple targeting RNAs [39]. These approaches assume that an activity defined by the majority of targeting RNAs reflects on-target effects, which may not always be the case. In addition, these approaches require a large number (usually 15 to 20) of targeting RNAs per gene, thus inapplicable to traditional siRNA or shRNA libraries that typically contain 4 to 6 targeting RNAs in each pool. In fact, the increased sequence complexity with a larger pool of targeting RNAs may induce additional off-target effects. We thus sought to utilize the data from primary and secondary screens with traditional arrayed siRNAs to filter out off-targets, again taking advantage of multiple functional readouts at each treatment condition.

As illustrated in Fig. 4c, we first identified siRNA pools that showed similar responses in pairwise comparison by requiring *R*>=0.6 (ref [40]). Because two genes may have related functions in a common biological pathway, more than one siRNA in their pools are expected to show similar responses to both of their pools in the secondary screen, as illustrated with *SNRPA1* and *SF3B1*, both being subunits of the U2 ribonucleoprotein particle (snRNP) (Fig. 4d, e). We further illustrated this with multiple core spliceosome components (Additional file 1: Fig. S4c). On the other hand, if a similar response resulted from certain off-targeting effects, one specific siRNA in a given siRNA pool would show sequence complementarity of consecutive 11nt or longer to the transcript targeted by the other siRNA pool (see Fig. 4f), as shown earlier when examining cross-reacting siRNAs [41]. Moreover, it would be the same siRNA that also induced the similar response in secondary screen, as exemplified with *FCHO1* and *SNRPB* (Fig. 4g). Here, *SNRPB* is a known core spliceosome component, whereas *FCHO1* is a gene functioning in early step of clathrin-mediated endocytosis [42], but without any documented role in regulated splicing, suggesting that the high *ζ* value generated by siFCHO1 resulted from its off-target effect on *SNRPB*. Based on these results, we propose a general strategy to eliminate potential off-target effects if a single siRNA in a given pool is responsible for (i) generating a similar functional response and (ii) showing a significant sequence complementarity to the transcript targeted by another siRNA pool. Using this strategy, we identified multiple siRNA pools that likely caused off-targets due to specific cross-reactions with well-established splicing regulators (Additional file 1: Fig. S4d).

We extended this analysis to all non-expressors on our screen and showed that filtering out those with identifiable off-targeting activities significantly improved the Screen Strength (Fig. 4a, from blue to red line). Furthermore, *ζ* scores may differ when different positive controls are used to generate the SMV. To evaluate this impact, we focused on high confidence hits after BP2 based on using repetitive siPTBP1 treatments as positive controls and found that > 90% of hits were identifiable with a different set of internal positive controls (see Additional file 1: Fig. S3a) to deduce a slightly different SVM line (Additional file 1: Fig. S4e-g), suggesting that slightly distinct positive controls only affect low-ranking candidates. Because of the ability to rank the hits, we were able to detect > 90% of the hits using siPTBP1-derived SVM based on the balance point alone without using any SVM (Additional file 1: Fig. S4f-g), although the ability to generate an SVM curve helps minimize the inclusion of low confidence hits.

Finally, we evaluated the performance of ZetaSuite on different numbers of functional readouts. Using true positives (siPTBP1) and high confidence hits based on using all AS readouts as the reference sets, we tested the ability of the *ζ* statistics to detect these “reference” genes using fewer readouts and found that the *ζ* statistics was indeed able to identify over 80% of these “reference” genes when the readout size reaches 200 or greater (Additional file 1: Fig. S4h). This information offers a general guide to designing future two-dimensional genome-wide screens.

### Application of ZetaSuite to understand core fitness genes in cancer cells

Having established the general framework of the *ζ* statistics with our in-house splicing screen data, we next sought to demonstrate its general applicability to other large-scale two-dimensional data. DRIVE [10] and DepMap [11] are representative of such data, designed to determine cancer dependencies. In these studies, pooled shRNAs were transduced into a large panel of cancer cell lines followed by deep sequencing to identify depleted shRNAs to identify genes critical for cancer cell survival. DRIVE tested more cell lines than DepMap (overlap=113, Additional file 1: Fig. S5a), whereas DepMap covered more genes than DRIVE (overlap=7,081, Additional file 1: Fig. S5b). Thus, as with our splicing screen dataset, the first dimension consists of individual shRNA treatments and the second corresponds to multiple functional readouts (different AS events vs different cell lines). Similar to our experimental design, DepMap selected a set of known essential genes (*n*=210, 43] as positive controls and used non-expressed genes (n=855) as negative controls, both serving as the benchmarks for validating the performance of ZetaSuite. We found that these controls are well separated based on t-distributed stochastic neighbor embedding (tSNE) [43] (Additional file 1: Fig. S5c).

For data analysis, DRIVE utilized RSA to rank-order hits and ATARiS to eliminate off-targeting shRNAs. A gene was considered essential if RSA>= − 3 in > 50% of the cell lines tested. In contrast, DepMap removed off-target effects with DEMETER and selected top hits showing 6 standard deviations (SD or σ) or greater in any cell line tested for further pathway analysis. As we demonstrated in treating our two-dimensional splicing screen data, an arbitrary cutoff would present a trade-off between sensitivity and specificity, and even with the most extreme cutoff like 6σ, experimental noise would still become accumulated with the increasing number of readouts from a screen. We thus tested the Screen Strength (SS) strategy in ZetaSuite to compare different screen results.

**Fig. 5:**
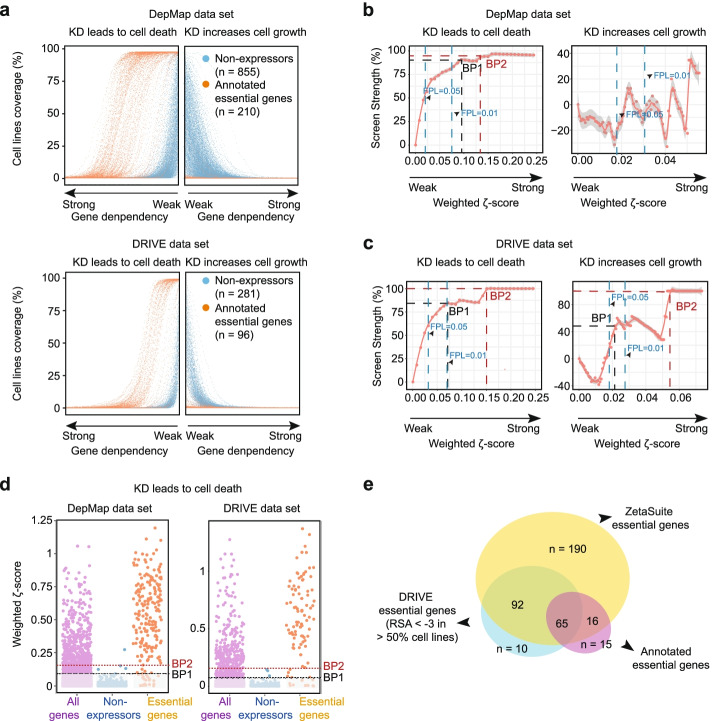
Application of ZetaSuite to mine core fitness genes in cancer cells. **a** At each gene dependency bin over a full range of gene dependency scores, the percentage of cell lines responsive to knockdown of individual annotated essential genes (orange dots) or non-expressed genes (blue dots) based on the DepMap (top) and DRIVE (bottom) datasets. **b**, **c** Screen Strength plot at different cutoffs for cancer dependency (left) or cancer checkpoint (right) deduced from the DepMap (**b**) or DRIVE (**c**) dataset. Because of scattered data, balance point could not be determined in the DepMap dataset. The two balance points (BP1 and BP2) in the DRIVE dataset are marked (**c**). Empirical FPL lines (0.05 and 0.01) are also indicated. **d** Hits for cancer dependency above the threshold defined by BP1 or BP2 based on the data from DepMap (left) or DRIVE (right). **e** Comparison of cancer dependencies deduced in the DRIVE project with those newly determined with ZetaSuite and previously annotated essential genes

We first processed the data from DepMap and DRIVE according to the ZetaSuite pipeline (see Additional file 1: Fig. S1b). Although DRIVE and DepMap mainly determined cancer dependencies by scoring depleted shRNAs, we took advantage of ZetaSuite to identify both depleted and enriched shRNAs. We utilized the processed data with potential off-target effects already removed and then plotted the data in both directions in the full range of cutoffs. As expected, positive controls and non-expressors were well separated in both datasets in the direction of cancer dependency (Fig. 5a), thus allowing us to calculate a weighted *ζ*-score for each tested gene, display the data in the SS plot, and detect two balance points (BP1 and BP2) in both datasets (Fig. 5b). Interestingly, we also detected enriched shRNAs, indicating that depletion of their target genes enhanced tumor cell growth, which we referred to as cancer checkpoints (see below). In the SS plot, we were unable to derive any balance point with the dataset of DepMap, likely due to scattered data from a relatively smaller number of cell lines surveyed (Fig. 5b), and with the dataset of DRIVE, we only used the most stringent cutoff at BP2 to select hits (Fig. 5c).

Based on the selected BP1 and BP2, the majority of positive controls were included in both datasets, suggesting that ZetaSuite-suggested cutoffs were able to encompass the majority of cancer dependencies, even at BP2 (Fig. 5d). This is in sharp contrast to alarmingly high error rates even at the stringent FDR cutoff with RSA, RIGER, MAGeCK, or CB^2^ (Fig. S5d). Since DepMap only focused on specific cancer dependencies by requiring 6σ, which is too stringent, we focused on comparison between ZetaSuite-identified hits and DRIVE-defined hits against the set of previously annotated essential genes [44]. Even at the cutoff based on BP2, ZetaSuite identified more hits than DRIVE hits (Fig. 5f), and moreover, none of the 10 DRIVE hits missed by ZetaSuite belong to the annotated essential genes (Fig. 5f, blue). Despite the significantly enlarged hit size, enriched Gene Ontology (GO) terms, KEGG pathways, and complexes annotated in the CORUM database [45] associated with newly identified hits were similar to those deduced earlier based on much more stringent cutoffs, with top-ranked terms linked to key housekeeping activities, such as DNA replication, splicing, cell cycle, RNA transport, and ribosome biogenesis (Additional file 1: Fig. S5e-g). In addition, those newly identified hits were largely anti-correlated with AGO2 expression and copy number variation (CNV) (Additional file 1: Fig. S5h), as reported earlier with the DRIVE dataset [10]. In contrast, 8 out of 10 hits identified by DRIVE but missed with ZetaSuite lacked such anti-correlation with either AGO2 expression (Additional file 1: Fig. S5g, top) or AGO2 CNV (Additional file 1: Fig. S5g, bottom). Together, these data demonstrated the effectiveness and objectiveness of ZetaSuite in identifying cancer dependencies from previous large-scale screen data.

### Biological insights into cancer dependency

**Fig. 6:**
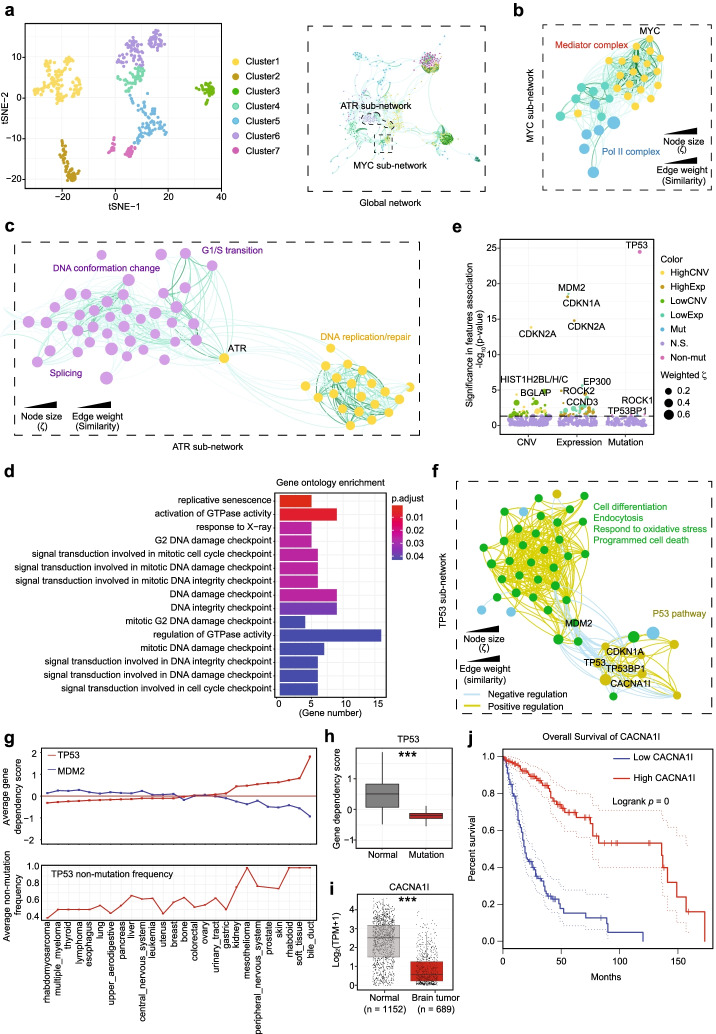
Biological insights from identified cancer dependencies. **a** Cluster (left) and global network (right) for cancer dependencies determined by ZetaSuite from the DRIVE dataset. **b**, **c***MYC*-associated sub-network, highlighting its connectivity to mediators and Pol II components (**b**) and *ATR* connectivity to sub-networks associated with genes involved in DNA conformation or DNA replication/repair (**c**). Colors correspond to different clusters defined in **a**. **d** Functionally enriched GO term biology pathways for cancer checkpoint hits based on the DRIVE dataset. Shown are top 15 GO terms with smallest adjust *p*-values. **e** The association of ZetaSuite-identified cancer dependencies with gene expression, copy number, and mutation features. For each gene, cancer cell lines were firstly ranked based on the levels of CNV or gene expression, and the cancer dependency scores were then compared between cell lines in top 25% versus bottom 25%. The *p*-value (*y*-axis) for each gene in this comparison was determined by Wilcox-test. In addition, for association analysis with mutations, cancer cell lines were divided in two groups with or without mutation for each gene. The cancer dependency scores were then compared between these two groups and the *p*-value (*y*-axis) in this comparison was determined by Wilcox-test. Some representative genes are highlighted in each feature group. Genes above the black dashed line have *p*-values < 0.05. **f***TP53*-associated sub-network. **g** Averaged dependency scores for *TP53* and *MDM2* (top) and *TP53* non-mutation frequency (bottom) in different cancer tissues. Tissues are ranked based on averaged *TP53* dependency scores. **h** The *TP53* gene dependencies in normal or mutated *TP53* cell lines. *** *p*<0.001 based on Wilcox-test. **i***CACNA1I* gene expression in normal brain tissues (based on the GTEx database) and brain tumors (based on the TCGA database). *** *p*<0.001 based on Wilcox-test. **j** Kaplan-Meier survival curves of brain tumor patients associated with high or low *CACNA1I* expression. The dashed lines indicate the 95% confidence intervals

The expanded list of cancer dependencies provided further insights into critical cancer development pathways compared to those already recognized from a previous analysis with the limited set of genes. For example, we deduced 7 clusters by t-SNE plotting and draw the global network based on regulation similarity based on similarities among different DRIVE cancer cells that passed the BP1 threshold (Fig. 6a). One of these gene networks was enriched with components of the transcription mediator complex and Pol II, all connected to the well-known oncogene *MYC* (Fig. 6b), consistent with the known function of MYC in transcriptional control [46]. Interestingly, MYC inhibition showed the most dramatic impact on rhabdoid cancer cells (Additional file 1: Fig. S6a), in agreement with a recent observation that MYC inhibition effectively restricted rhabdoid tumor growth in vivo [47]. In this MYC dependency plot, significant *MYC* dependency was noted in multiple myeloma (MM) cancer cells, in line with frequent 8q24 translocation that leads to MYC overexpression in MM cancers [48].

To further demonstrate the utility of ZetaSuite in analyzing the DRIVE and DepMap datasets to mine important cancer pathways, we analyzed two separate clusters connected by *ATR*, a key regulator of genotoxic stress. One cluster includes various genes involved in G1/S transition and modulation of DNA topology and the other encompasses genes critical for DNA replication/repair (Fig. 6c). This is consistent with the existing literature on the function of ATR in connecting genotoxic stress to cell cycle control [49]. Notably, several splicing regulators (i.e., *SRSF1* and *SRSF2*) are present in these clusters, both being implicated in inducing aberrant R loops that led to ATR activation [50]. This has been suggested as a key mechanism underlying Myelodysplastic Syndromes (MDS), a pre-leukemia that has the propensity to rapidly progress to acute myeloid leukemia (AML), thus explaining greater ATR dependency in leukemia than most other cancer types (Additional file 1: Fig. S6b).

### Genes involved in cancer checkpoint

One of the most significant advances in further mining the DRIVE dataset with ZetaSuite is the discovery of genes whose depletion appears to promote tumor growth. Strikingly, GO term analysis revealed that the vast majority of these genes were involved in DNA checkpoint control (Fig. 6d). Previously, genes involved in cancer dependencies were cross analyzed with copy number variation (CNV), gene expression, or mutation frequencies, revealing their association with low CNV and low expression, which has been referred to as CYCLOPS genes [51]. We further confirmed this with ZetaSuite-identified cancer dependencies (Additional file 1: Fig. S6c). We next extended the analysis to cancer checkpoint genes and identified 9 major clusters (Fig. 6e). Contrary to core fitness genes, however, much fewer cancer checkpoint genes were associated with CNV, altered expression, or mutation in DRIVE cell lines.

Several typical tumor suppressors were identified as strong cancer checkpoints in this feature association analysis, including *TP53* (encoding for p53) [52] and its transcription target *CDKN2A* (encoding for the cell cycle inhibitor p16) [53] and *CDKN1A* (encoding for the cell cycle inhibitor p21) [54]. *MDM2*, an E3 ligase for p53, was also identified as a cancer checkpoint gene (Fig. 6e) and the similarity network clearly reflects the antagonizing function between *TP53* and *MDM2* (Fig. 6f). In fact, while wildtype *TP53* always gave rise to a positive dependency score, reflecting its tumor suppressor function, mutant *TP53* produced a negative cancer dependency score, indicating its oncogenic role in those tumor cells (Fig. 6g, h), in agreement with the established roles of wildtype and mutant p53 in tumorigenesis [55]. Most interestingly, as exemplified with *MDM2*, multiple cancer checkpoint genes were also linked to either low CNV or low expression (see Fig. 6e), suggesting that the CYCLOPS phenomenon applies to some key cancer checkpoints as well. *MDM2* was also connected to a cluster of genes functioning in cell differentiation, endocytosis, cell death, and response to oxidative stress, consistent with the role of MDM2 in regulating the transition from proliferation to differentiation [56] and in the cellular response to oxidative stress [57].

In the elucidated p53 subnetwork, *TP53BP1* and *ATM* activate *TP53*, which in turn activates *CDNK1A* (Fig. 6f). Besides these known functional connections, we also identified various genes without prior connection to the p53 pathway, such as *PCOLCE* and *CACNA1I*. As an extracellular matrix protein and a major regulator of fibrillar collagen biosynthesis, disruption of *PCOLCE* had been reported to induce cell growth in cultured fibroblasts, suggesting a role in cell proliferation control [58]. *CACNA1I*, a gene involving controlling voltage-gated calcium channels, was significantly down-regulated in brain tumors compared to surrounding normal tissues (Fig. 6i), and patients with low *CACNA1I* expression were associated with poor prognosis based on the TCGA database (Fig. 6j). The newly discovered connection of this and other critical genes with the p53 pathway would fuel future studies on tumorigenesis.

Last, but not least, further analysis of the newly identified cancer checkpoints revealed several major regulatory gene networks based on their similarities among different DRIVE cell lines (Additional file 1: Fig. S6e). Besides those critical points of cell aging, such as *TP53*, *CDKN2A*, *BGLAP*, and *CDKN1A*, as described above, we also noted gene networks for phosphorylation regulation (e.g., *MAP3K9*, *TAOK1*, *ROCK1/2*), GTPase activities (e.g., *EPHA5*, *TBC1D3D*, *RND3*), and DNA packaging (e.g., *HIST1H2BN*, *HIST1H2BL/H/C*). These findings not only support the documented roles of specific MAPK and Rho GTPase pathways in tumorigenesis [59, 60], but also raise a new paradigm regarding how DNA packaging proteins may promote tumor growth. Collectively, this functional connectivity map provides critical insights into the involvement of an elaborated gene network in checkpoint control, which may be critical for long-term cell survival, even among cancer cells.

### Using ZetaSuite to QC single-cell sequencing data

Single-cell transcriptomics analysis has become a powerful tool to characterize cellular heterogeneity in specific biological contexts. A challenge in these studies is how to differentiate high-quality cells from damaged ones, which has the potential to severely compromise specific conclusions reached. Three independent quality control (QC) metrics, nCount, nFeature, and %mt (percentage of mitochondrial transcripts) have been introduced to evaluate the quality of individual cells [61, 62], but the popular approaches with a defined threshold, such as CellRanger and EmptyDrops [21], still mainly rely on one of these metrics (nCount) to QC sequenced cells. Therefore, it would be desirable to use more than one independent metric. ZetaSuite is ideally suited for this purpose by plotting the number of genes (*y*-axis, reflecting nFeature) counted at each expression bin (x-axis, reflecting nCount) in the *ζ* plot, thus providing a *ζ* score for each sequenced cell.

**Fig. 7:**
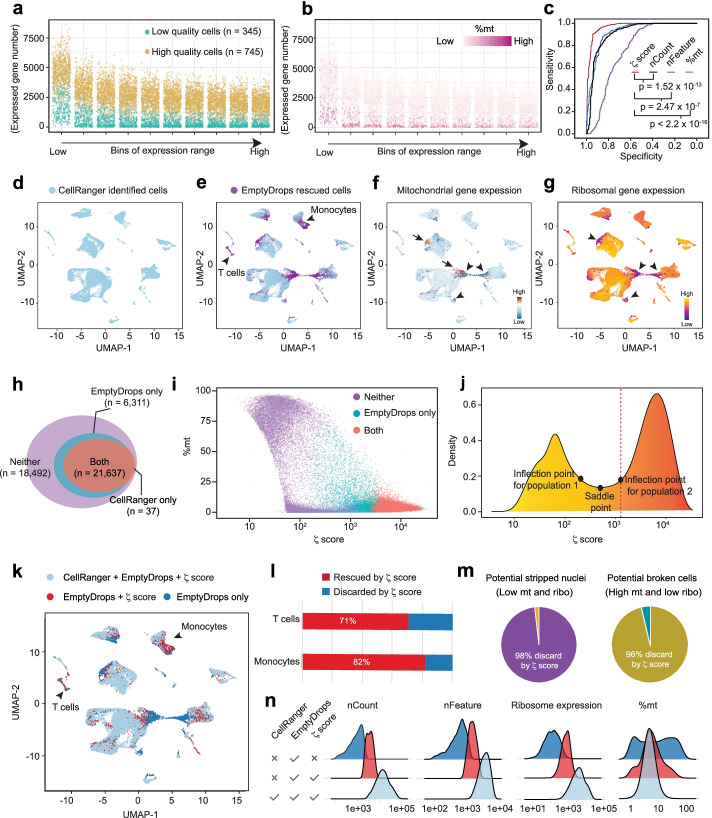
Application of ZetaSuite to single-cell transcriptomics. **a***ζ*-plot at each gene count bin over a full range of gene counts. Raw counts of each gene are plotted against the number of different genes detected. High-quality (orange dots) or low-quality (blue dots) cells are based on the benchmark dataset (E-GEOD-48968). **b** Same *ζ*-plot with cells colored based on %mt. **c** ROC curves deduced using different metrics. The *p*-values are calculated by plot.roc in pROC R package with default parameters. **d** UMAP of cells based on the CellRanger cutoff. **e–g** UMAP of cells based on the cutoff by CellRanger or EmptyDrops software. Colors were labeled by detection software (**e**), expression of mitochondrial transcripts (**f**), or levels of ribosomal RNA (**g**). **h** Cells’ number detected by both CellRanger and EmptyDrops (red, both), missed by both software (purple, neither), or rescued by EmptyDrops (blue). **i** Plotting *ζ*-scores of individual cells against their %mt. Colors label cells as in **h**. **j** Same as (i) except plotting the cell density in *y*-axis. **k** UMAP of cells that meet the cutoffs of CellRanger, EmptyDrops, and ZetaSuite (light blue) in comparison with those that meet the cutoffs of EmptyDrops and ZetaSuite (red) or the cutoff of only EmptyDrops (dark blue). Colors were labeled by detection software. **i** Percentage of T cells or monocytes identified by EmptyDrops and ZetaSuite (red) or only EmptyDrops (blue). **m** Percentages of stripped nuclei (left, characterized by both low %mt and ribosome expression) or broken cells (right, associated with high %mt but low ribosome expression due to selective leakage of cytoplasmic mRNAs from broken membrane) identified by EmptyDrops but discarded by ZetaSuite. **n** Ridgeline plot showing the distribution of nCount, nFeature, %mt, and ribosome expression for cells detected only by EmptyDrops, by both EmptyDrops and ZetaSuite, or by all three software, showing the ability of ZetaSuite to rescue high-quality cells missed by CellRanger while filter out damaged cells also rescued by EmptyDrops

To demonstrate this approach, we utilized a benchmark dataset in which individual sequenced cells were visually inspected by microscopy to segregate them into high-quality or low-quality class [63]. We divided transcript counts into 10 expression bins and quantified the number of distinct genes covered within each bin, thus generating a *ζ* plot for all sequenced cells (Fig. 7a). By color-labeling each cell pre-determined as high-quality (yellow) or low-quality (cyan) in this *ζ* plot, we found that all high-quality cells are well separated from low-quality ones (Fig. 7a). We also color-labeled each cell in the same *ζ* plot according to different ranges of %mt, observing that those with exceptionally high %mt, which likely result from broken cells, are all distributed at bottom (low nFeature values across all nCount bins), thus giving rise to small *ζ* values (Fig. 7b). Additionally, receiver operating characteristic (ROC) curves showed that the *ζ* statistics-based approach significantly outperformed nCount-, nFeature-, and %mt-based QC strategies (Fig. 7c).

To further test the *ζ* statistics-based QC strategy, we also generated the *ζ* plot based on another benchmark dataset, which additionally annotated low-quality cells into broken cells or empty droplets by microscopy [64]. Despite limited cells in this dataset, which gives rise to a significantly scatted plot, it is still evident that both broken cells and empty droplets are effectively segregated from high-quality cells (Additional file 1: Fig. S7a). This is further evidenced by comparing individual cells scored with different metrics. While all metrics except %mt showed a similar ability to segregate high-quality cells from empty droplets, the *ζ* metric demonstrated much improved efficiency in differentiating high-quality cells from broken cells, especially in comparison with nFeature (Additional file 1: Fig. S7b), which is further supported by comparing ROC curves generated by *ζ* or nFeature metric (Additional file 1: Fig. S7c).

### Application of ZetaSuite to maximize the power of single-cell transcriptomics

To demonstrate the power of ZetaSuite in analyzing single-cell transcriptomics, we utilized a scRNA-seq dataset generated from placenta [65] that has been analyzed with CellRanger and later used to develop EmptyDrops. As demonstrated earlier, EmptyDrops was able to “rescue” two critical cell populations (T cells and monocytes) missed by CellRanger (Fig. 7d, e and Additional file 1: Fig. S7d-e). However, this gain is at the expense of including other cells with abnormal %mt (Fig. 7f, red for broken cells indicated by arrows; dark blue for stripped nuclei pointed by arrowheads) and low ribosomal RNA (Fig. 7g, purple). For comparison, we calculated *ζ* scores for all sequenced cells, including those below the cutoff by CellRanger and EmptyDrops (Fig. 7h, neither), rescued by EmptyDrops (Fig. 7h, EmptyDrops only), and identified by both CellRanger and EmptyDrops (Fig. 7h, both). Interestingly, the plot of %mt vs *ζ* score revealed that cells with high *ζ* scores include those commonly identified with CellRanger and EmptyDrops as well as about half of EmptyDrops-rescued ones while the remaining cells were most associated with abnormal %mt values (Fig. 7i). The density plot of *ζ*-scored cells clearly showed two cell populations, which allowed us to make a standardized cutoff based on the reflection point of the second population to minimize the contamination of the first population (Fig. 7j).

We next returned to the UMAP plot to locate EmptyDrops-rescued cells with (red) or without (dark blue) support by ZetaSuite (Fig. 7k). We were able to retain ~3/4 of EmptyDrops-rescued T cells and monocytes (Fig. 7l) yet eliminate the vast majority of broken cells and stripped nuclei (Fig. 7m). Finally, by displaying the distribution of cells quantified by each of the 4 metrics (Fig. 7n), it is clear that cells satisfying all three methods (methods implemented in CellRanger, EmptyDrops, and ZetaSuite) showed the highest range in nCount, nFeature, and ribosome RNA expression as well as balanced %mt (Fig. 7n, light blue). In comparison, among EmptyDrops-rescued cells, ZetaSuite retained cells with biological meanings (Fig. 7n, red) while eliminated broken cells and stripped nuclei (Fig. 7n, dark blue). Together, these data demonstrate the power of the *ζ* statistics in combining the benefits of both CellRanger and EmptyDrops without compromising the data quality in single-cell transcriptomics analysis.

## Discussion

The increasing power and decreasing cost of deep sequencing technologies have enabled multi-dimensional analyses of gene expression. By coupling high-throughput screening with high-throughput sequencing (HTS^2^), it is possible to utilize a specific set of genes as a surrogate for defined cellular activities in chemical and genomic screens [8, 9]. Through monitoring hundreds or even thousands of functional readouts, such “ultrahigh-content” screens offer numerous advantages over traditional one-dimensional screens, including the ability to deduce gene networks and the feasibility to perform a drug screen without relying on a pre-defined druggable target. More recently, we have extended the HTS^2^ approach to a genome-wide screen to identify global splicing regulators by scoring hundreds of alternative splicing events, illustrating the ability to adapt two-dimensional screens to study different paradigms in regulated gene expression.

This added dimension also requires a concerted effort in developing suitable statistics for data analysis. In the current work, we introduce a newly developed *ζ* statistics, and by using our in-house HTS^2^ data designed to identify global splicing regulators, we demonstrate that *ζ* statistics outperforms the existing strategies based on hit ranking and aggregation_,_ such as RSA [23], RIGER [24], MAGeCK [25], and CB^2^ [26]. Additionally, we note that these existing methods rely on a null hypothesis that most screened genes are non-hits, thus not suitable for analyzing data from secondary screens or using pre-selected candidates. In contrast, the *ζ* statistics can be broadly used to process two-dimensional data, which requires a significant number of negative controls. As demonstrated in our current work, non-expressed genes provide a large set of internal negative controls. In ZetaSuite, we also introduce the Screen Strength to measure the success of a given screen and to compare between screens.

Off-target effects represent a major problem in genome-wide screens with siRNAs, shRNAs, or sgRNAs. To reduce the impact of off-target effects, one strategy is to increase the number of targeting RNAs (up to 50 per gene) against each gene [66]. Multiple algorithms have been developed to remove potential off-target effects. For example, ATARiS was developed based on the assumption that multiple on-targeting RNAs would give rise to similar results while off-targeting RNAs would each cause a distinct non-specific effect [39]. This assumption had the potential to retain off-targeting hits when multiple targeting RNAs caused similar non-specific effects, for instance, due to induced cellular stress. In comparison, DEMETER [11] or its recently refined version DEMETER2 (ref [67]) filtered out off-targeting effects based on the assumption that off-targets likely result from the sequences in the “seed” region to cause microRNA-like effects on other genes. Common seed analysis is another strategy to identify off-targeting siRNAs according to the same assumption as DEMETER based on the assumption that the seed sequences would be statistically overrepresented in active siRNAs in off-target effects as compared to inactive siRNAs [68]. This assumption might not be reliable because of numerous exceptions to the “seed rule” and various miRNA-like effects induced by sequences outside the seed region [69]. In contrast to the existing approaches, ZetaSuite eliminates off-targets based on two criteria, one on the functional similarity and the other on the sequence complementarity between a targeting RNA and a potential off-targeted transcript. Furthermore, by leveraging the results from the secondary screen, we found that a single siRNA in a pool is often responsible for the off-targeting effect of that pool and the same siRNA also shows the complementary sequence to the predicted off-target. Therefore, besides removing off-targeting effects, ZetaSuite may also help identify genes that tend to be off-targeted, thereby aiding in siRNA library design similar to GESS [70, 71]. We further note that ZetaSuite could be coupled with SIGNAL [72], an algorithm for prioritizing selected hits according to the information on functional networks and pathways.

We further demonstrate the utility of ZetaSuite by processing the large-scale data from public DRIVE and DepMap cancer dependency projects. Prior efforts in analyzing these datasets had been primarily focused on cancer dependencies, revealing various gene networks critical for cancer cell survival. DRIVE defined cancer dependency by requiring RSA>= − 3 on > 50% of cell lines surveyed while DepMap paid particular attention to hits with 6σ or greater. These definitions appeared to be arbitrary, and in the case of DepMap, the cutoff was unnecessarily too stringent without fully exploring the information contained in such large-scale datasets. By revisiting these data with ZetaSuite, we elevated the number of clear cancer dependencies by several folds, leading to the elucidation of multiple new gene networks contributed by some well-established oncogenes and tumor suppressors, such as *MYC*, *ATR*, and *TP53*. These discoveries potentiate further dissection of fundamental oncogenic pathways. The most important discovery made by re-analyzing the DRIVE dataset is the identification of genes whose depletion appears to accelerate cancer cell proliferation, at least transiently during the treatment period. Strikingly, most hit functions in various DNA checkpoint pathways, which we refer to as cancer checkpoint. Such depletion-induced cell proliferation might allow cancer cells to temporally escape DNA checkpoint control, indicating that various cancer cells need to maintain a very active program to protect their unstable genomes from becoming further deteriorated. In this regard, the exposure to these new cancer vulnerabilities might aid in the development of new cancer therapies, as exemplified by using ATR inhibitors to treat MDS [73].

We also demonstrate the utility of ZetaSuite in addressing a pressing problem in analyzing single-cell transcriptomics, which is to maximally retain high-quality cells and remove damaged ones. This problem is also related to the problem associated with using simple statistics to make an arbitrary cutoff, as many real hits may escape detection with a stringent cutoff but many false positives would be retained with a loose cutoff. In single-cell transcriptomics analysis, the state-of-art approach is to use nCount to differentiate high-quality cells from damaged ones, as with CellRanger, but the recently developed EmptyDrops clearly exposed the weakness of CellRanger by showing important cell populations missed [21]. However, EmptyDrops appears to introduce other unwanted artifacts. We have now used ZetaSuite to address this trade-off by incorporating critical features of both nCount and nFeature. Using benchmarked datasets, we demonstrated that the newly developed *ζ* statistics can maximally segregate high-quality cells from damaged ones while minimize unwanted artifacts. These studies, coupled with mining DepMap and DRIVE datasets, showcase the power of ZetaSuite in processing multi-dimensional high-throughput data to reveal critical biological meanings embedded in those large-scale datasets.

## Conclusions

The increasing power of deep sequencing has enabled the generation of high throughput data under many different conditions, representing a second dimension of high-throughput data. However, the existing bioinformatics tools are largely designed to process one-dimensional high-throughput datasets, which we demonstrate to cause noise accumulation when the scale of the second dimension is significantly increased. We have thus developed a new statistics called Zeta and associated software package ZetaSuite for processing two-dimensional high-throughput datasets and demonstrated that ZetaSuite outperforms current benchmark statistical models, leading to novel biological insights and illustrating the broad applicability of ZetaSuite in diverse functional genomics studies.

## Methods

ZetaSuite is designed to address challenges in analyzing two-dimensional high-throughput data. Additional file 1: Fig. S1b provides an overview of the flow chart, as individually detailed below.

### ZetaSuite part 1

#### Data preprocessing

Before running the main ZetaSuite procedure, raw data are first filtered to remove low-quality samples (columns) and readouts (rows) in the data matrix to minimize false positives. The default threshold is set to remove a row or a column if the number of drop-outs (missing values; in our in-house dataset, the ratios are used as input and the ratio is missing if one of the mRNA isoforms is undetectable) is larger than the value of *Q*_3_+3*(*Q*_3_-*Q*_1_) where *Q*_1_ and *Q*_3_ are lower and upper quartile, respectively. The remaining data are processed with the KNN-based method to estimate the missing values with the parameter *k*=10.

### ZetaSuite part 2

#### QC evaluation

Quality control (QC) is a critical step in evaluating the experiment design. For all two-dimension high-throughput data, t-SNE plot [43] is first used to evaluate whether features are sufficient to separate positive and negative controls. The SSMD score [15] is further generated for each readout to evaluate the percentage of high-quality readouts. In our case, the data will be further processed if > 5% of reads are of the SSMD score > 2.

#### Conversion of input matrix to Z-score matrix

After data pre-processing, the initial input matrix is arranged in N x M dimension, where each row contains individual functional readouts against a siRNA pool and each column corresponds to individually siRNA pools tested on a given functional readout. Readouts in each column may be thus considered as the data from a one-dimensional screen (many-to-one), and thus, the typical *Z* statistics can be used to evaluate the relative function of individual genes in such column. The conversion is repeated on all columns, thereby converting the raw activity matrix into a *Z*-score matrix. Suppose *N*_ij_ are the values in the original matrix i (1≤ i ≤ N siRNA pool) row and j (1≤ j ≤ M readout) column, then

$${Z}_{ij}=\frac{N_{ij}-{\mu}_j}{\sigma_j}$$

where *μ*_*j*_ and *σ*_*j*_ are the mean and standard deviation of negative control samples in column j.

#### Generation of Zeta plot

The *x*-axis in the Zeta plot shows a series of *Z*-score cutoffs in two directions (in our case, induced exon skipping in the positive direction and inclusion in the negative direction), and the *y*-axis is the percentage of readouts survived at a given Z-score cutoff over the total scored readouts.

To generate this plot, the range of *Z*-scores is first determined by ranking the absolute value of total *Z*_ij_ (*Z*-score value in row i and column j) from the smallest to the largest (|*Z*_1_|, |*Z*_2_|, …∣*Z*_*k* − 1_∣, ∣*Z*_*k*_∣, |*Z*_*k* + 1_∣, …,∣*Z*_*N* × *M*_∣_,_where ∣*Z*_*k* − 1_∣≤|*Z*_*k*_∣≤|*Z*_*k* + 1_∣ and k here is the rank number). To exclude insignificant changes that may result from experimental noise (choose |Z|=2 as cutoff. In standard normal distribution, using |Z|>2 as a rejection region, the corresponding *p*-value is < 0.05), *Z*-score cutoffs are selected in the range of [-∣*Z*_⌊*N* × *M* × 0.999⌋_∣, -2] in the negative direction and [2, ∣ *Z*_⌊*N* × *M* × 0.999⌋_∣,] in the positive direction. The *Z*-score range in both directions is next divided into 100 bins (B = (*b*_1,_*b*_2_, …, *b*_*i*_, …, *b*_100_), where *b*_*i*_ = [*Z*_*min*_ + (*Z*_*max*_ − *Z*_*min*_) × (*i* − 1)/100, *Z*_*min*_ + (*Z*_*max*_ − *Z*_*min*_) × (*i*)/100]; *Z*_*max*_ is either -2 or ∣*Z*_⌊*N* × *M* × 0.999⌋_∣ and *Z*_*min*_ is either -∣*Z*_⌊*N* × *M* × 0.999⌋_∣ or 2. Next, for each siRNA pool, the percentage of readouts scored above the *Z*-score cutoff in each bin is determined.

#### Calculation of ζ score and weighted ζ score

When a screen includes a large number of both negative and positive controls, these controls are all displayed in a Zeta plot. Radial kernel SVM is next constructed to maximally separate positives from negatives in the prior defined *Z*-score range using e1071 packages of R. To avoid overfitting, it is important to use an independent dataset, such as non-expressors as internal negative controls, to confirm the deduced SVM. To provide a value to represent the regulatory function of gene i that generates a curve above the SVM curve, the area between the two curves is calculated as the Zeta score (*ζ* score) for this gene. To calculate the total area, we first divide the *Z*-score range into 100 bins, and at each bin, we determine the number of readouts that show significant changes above the *Z*-score cutoff at the bin for each siRNA-targeted gene and then divide this number with the total number of measured readouts. After subtracting the background percentage (based on the SVM curve), we obtain the increased percentage of readouts that show significant changes. To highlight hits scored at higher *Z*-score bins, the area in each bin is multiplied by the value of the Z-score in such bin and all adjusted areas are summed to give rise to the final weighted *ζ* score for each gene:

$${\zeta}_i=\sum_{m={Z}_{min}}^{Z_{max}}{Area}_m\times m$$

where the *Area*_*m*_ is the area in the specific *bin*_*m*_:

$${Area}_m=\left\{\begin{array}{c}\frac{\left(\left({P}_{m+1}+{P}_m\right)-\left({S}_{m+1}+{S}_m\right)\right)\ast step}{2}; if\ \left({P}_{m+1}+{P}_m\right)>\left({S}_{m+1}+{S}_m\right)\\ {}\ 0; if\ \left({P}_{m+1}+{P}_m\right)\le \left({S}_{m+1}+{S}_m\right)\ \end{array}\right.$$

where the *P*_*m*_ and *P*_*m* + 1_ are the *y*-axis values of gene i in the Zeta plot whereas *S*_*m*_ *and S*_*m* + 1_ are the *y*-axis values on the SVM curve, both at *bin*_*m*_ and *bin*_*m* + 1_; step is the bin size which equals to (*Z*_*max*_ − *Z*_*min*_)/100.

With certain screens without any positive controls, it will be impossible to generate an SVM curve to help eliminate experimental noise. In these applications, it is still possible to calculate a *ζ* score for each gene by determining the *Area*_*m*_ under the gene-specific curve at *bin*_*m*_:

$${Area}_m={\displaystyle \begin{array}{c}\frac{\left({P}_{m+1}+{P}_m\right)\ast step}{2}\ \\ {}\ \end{array}}$$

where the *P*_*m*_, *P*_*m* + 1_ and step are the same as those with the area with an SVM curve.

Although ζ scores are separately generated in our application to quantify the contribution of a given gene to exon inclusion or skipping, the absolute values of these ζ scores may also be summed to reflect the global activity of such gene in regulated splicing. ZetaSuite generates this summed value as the default data output unless users select “-c no” to separately generate two ζ scores in opposition directions.

#### Screen Strength and determination of the threshold for hit selection

The ζ scores can be used to rank genes and the next important step is to define a suitable cutoff to define hits at different confidence levels. For this purpose, the concept of Screen Strength is first introduced:

$$SS=1-\frac{aFDR}{bFDR}$$

where aFDR (apparent FDR) is the number of non-expressors identified at hits divided by the total number of hits and bFDR (baseline FDR) is the total number of non-expressors divided by all screened genes.

Based the definition of SS, the SS values would be progressively elevated with increasing cutoff stringency. A larger SS would indicate a lower false discovery rate but with a reduced number of hits. To address this trade-off, we suggest defining the balance point (BP) in the Screen Strength plot as follows: *ζ* scores are first divided into 100 even bins from the smallest to the largest and the SS value is determined at each bin. Connecting individual SS values then generates a simulated SS curve, based on which to deduce individual BPs. In order to directly reflect the error rate of selected hits according to the BPs, several empirical false positive levels (0.05, 0.01) are also provided in our SS plot. Users may choose one or multiple BPs to identify hits at different SS intervals according to their error tolerance. A successful screen is associated with a progressive increase in SS values compared to random draw.

### ZetaSuite part 3

#### Removing off-targeting hits

In the genome-wide screening, siRNAs are designed to specifically degrade mRNA transcripts of complementary sequences to reduce the expression of gene products. In practice, these reagents exhibit a variable degree of suppression of the targeted gene and may also suppress genes other than the intended target. The reagent’s phenotypic effects resulting from the suppression of unintended genes are called off-target effects. The reason for off-targets is due to the part-sequence complementary such as the microRNA-like off-targeting. And the consequence of off-targets is the phenotype or the effects on the readouts mainly due to off-targeting to a function gene. Multiple methods have developed to deal with the off-targeting problem based on the reason (refer DEMETER2, Common Seed Analysis) and consequence (refer ATARiS). Different from the many-to-one traditional screening data, the HTS^2^ can better evaluate the phenotype consistency by comparing the similarity effects on all the readouts. Based on these conditions, we define the off-targeting hits by combining the off-targeting reason and consequence together via comparing the hits with user-defined well-known genes or total-defined hits: (1) the off-targeting genes should have one of the targeting RNAs targeted to the well function genes (at least 11nt complementary sequence in the targeting RNA), and (2) they should show high similarity on the readouts’ effects with targeted well function genes (Pearson correlation score > 0.6).

#### Functional interpretation of identified hits

ZetaSuite combines Gene Ontology and CORUM databases [46] to infer functions. We use ClusterProfiler [74] to enrich hits on GO terms and present top 15 GO terms with lowest adjust *p*-values. To annotate hits to CORUM complexes, we present top 15 complexes associated with the highest number of hits. If less than 15 complexes are enriched, we require at least 3 hits to retain a complex.

#### Network construction

The SC3 method [75] is modified to use the absolute values of Spearman and Pearson correlation scores to calculate the distance matrix, which is next used to perform clustering. After SC3 analysis, each gene pair receives a consensus score, which measures the regulation strength. Edge weights reflect consensus scores and edge types indicate correlation or anti-correlation between gene-gene similarities. Nodes in the network represent the hits identified by the ZetaSuite pipeline and the size of each node is proportional to the *ζ* score. Node colors correspond to the clusters calculated with SC3 and cluster number is according to the total within-cluster sum of the square “elbow” site. The resultant hit networks are visualized with Gephi by using a Yifan Hu Proportional layout [76]. Disconnected nodes are trimmed from the graph before generating the plots.

### Other experimental procedures

#### Testing the multiple testing correction methods on error rate reduction

The multiple testing correction methods, like FDR and Bonferroni correction, are frequently used to reduce error accumulation in multiple hypothesis testing. However, it can only be used to deal with the data from one-dimensional screens but is not suitable for screens of two or multiple dimensions. To further test this, a common cutoff is *Z*-score>=3 or <= − 3, and thus, the estimated false positive level (*p*-value) is below 0.01, meaning that for each readout, a given siRNA has a 1% chance to be identified as a false positive hit. For all conditions, ~15,000 tests for each readout are performed and using the most stringent Bonferroni correction, we obtain a corrected *p*-value of 0.01/15000=6.67 × 10^−7^ and a corresponding *Z*-score=4.97. Now using *Z*-score=4.97 as the corrected cutoff to choose hits, we find that the false positive level is still as high as 24.9%. Instead of choosing an empirical *Z*-score as a threshold, we also use Gumbel distribution to estimate the *p*-value for each siRNA pool. In this procedure, the maximum absolute *Z*-score for each siRNA pool is firstly extracted. Then, R package evd is used to estimate the parameters of Gumbel distribution. Finally, the *p*-values for all screened siRNA pools in the Gumbel distribution are corrected by Bonferroni correction. We find that the threshold is *Z*-score=18.442 with a corresponding Bonferroni adjusted *p*-value=0.01. At this condition, all positive controls are filtered out, and the FDR value is as high as 100%. The FDR value is still as high as 94.9% even if we change Bonferroni correction to a more lenient correction, FDR correction. In these analyses, the dominance of random noises that are of high *Z*-score values likely results in the failure in selecting a threshold based on the Gumbel distribution. We conclude that such canonical multiple testing correction methods are not sufficient to reduce the accumulation of errors with increasing readouts in two-dimensional high-throughput screens.

#### Evaluating the optional number of functional readouts in two-dimensional screen

Positive controls and high-confidence hits, the latter of which are defined based on total readouts, are used as references in our evaluation. The number of readouts is progressively down-samples to 50, 100, 150, 200, 250, and 300 using R Sample function without replacement and each specific number of down-sampled readouts is replicated 3 times. Down-sampled matrixes are processed using the same ZetaSuite pipeline. Hits from down-sampled matrixes are used to determine the percentage of the hits over the reference sets.

#### Analysis of the splicing screen data with RIGER

RIGER is originally developed to identify essential genes in genome-scale shRNA screens [24]. In RIGER, the signal-to-noise ratio is entered as input, which is now replaced with the *Z*-scores for individual alternative splicing readouts. The data are then processed with the latest version of RIGER (2.0.2) from the website as provided in the source table above. Default RIGER parameters are used in all steps, except that the number of permutations is set to 100,000 to obtain a more precise *p*-value for each pool of siRNAs. The FDR is computed from the empirical permutation p-values using the Benjamini-Hochberg procedure. This enables the ranking of siRNA pools by FDR.

#### Analysis of splicing screen data with RSA

RSA is a probability-based method to identify hits, requiring data generated with multiple targeting siRNAs against each gene [23]. In RSA, fold-changes of treated over control samples are entered as input. In our application, the inputs are fold-changes of the splicing ratio of a given alternative splicing event in a siRNA pool-treated well divided by the averaged splicing ratio from NS-mix treated wells. The entered data are processed with the latest RSA software, as specified in the source table above. The following parameters -l 0.2 -u 0.8 and -l 1.2 -u 2.0 are used to select hits for induced exon inclusion and skipping, respectively.

#### Analysis of splicing screen data with MAGeCK

MAGeCK is a statistical method designed to quantify the collective activity of multiple siRNAs against each gene by using the robust rank aggregation (RRA) algorithm [25]. In order to meet the MAGeCK input requirement, each *Z*-score in the ZetaSuite input matrix is first converted to *p*-value. The input data are processed with the modified RRA algorithm, as in MAGeCK, with default parameters.

#### Analysis of splicing screen data with CB^2^

CB^2^ is a method using the Fisher’s combined probability test to combine the p-values of sgRNAs for a targeted gene after comparing the difference in functions of individual sgRNA using modified Student’s *t*-test [26]. In order to meet the CB^2^ input requirement, each *Z*-score in the ZetaSuite input matrix is first converted to a *p*-value. The input data are processed with Fisher’s combined probability test, as in CB^2^, with default parameters.

#### Processing DRIVE and DepMap cancer dependency datasets

The DRIVE and DepMap data already processed with DEMETER2 are downloaded from https://depmap.org/portal/download/. DepMap generated 3 independent datasets. In order to avoid experimental variations in different datasets, only the biggest DepMap dataset is selected for current analysis, which includes 285 cancer cell lines across approximately 100k shRNAs. ZetaSuite is applied to this dataset to calculate weighted *ζ*-scores with the parameters -z no –svm no and -c no. The downloaded data are provided as input for RSA and RIGER analysis. To meet the input requirement of MAGeCK and CB^2^, the processed data are transferred to percentile ranks and then processed by each software with default parameters.

#### Feature association analysis on cancer dependencies and checkpoints

To analysis association with CNV or gene expression, cancer cell lines are ranked based on the levels of CNV in a given gene or expression of the gene. Cancer dependency scores are next compared between cell lines in top 25% versus bottom 25% and Wilcox-test is performed to determine the *p*-value for the gene. To analysis association with mutations, cancer cell lines are divided in two groups with or without mutation in each gene. The cancer dependency scores are next compared between these two groups and Wilcox-test is performed to generate the *p*-value for the gene.

#### Processing single-cell datasets

Single-cell RNA-seq (scRNA-seq) generates gene expression in one dimension across a set of single cells in the second dimension, thus suitable for processing with the ZetaSuite pipeline. To calculate the *ζ* score for each sequenced cell, the raw counts of individual detected genes are divided into bins in *x*-axis, equivalent to individual *Z*-scores in our splicing screen. This reflects the feature of nCount. At each expression bin, the number of genes scored above such bin is plotted in *y*-axis, thus reflecting nFeature. If the data contain well-annotated negative controls, a SVM curve can be generated; otherwise, the area under the connected line for each cell can be calculated, which can be used to rank-order individual sequenced cells. In scRNA-seq analysis, it is unnecessary to generate a weighted *ζ* score for each cell.

The raw sequencing reads from two benchmark datasets are respectively downloaded (E-GEOD-48968 and PRJEB4039) from ArrayExpress Archive [77] and European Nucleotide Archive [78]. To calculate the efficiency, raw sequencing reads of all broken/empty cells and randomly selected 90 high-quality cells for the second benchmark dataset are also downloaded. Sequence reads are mapped to the Mus musculus genome (Ensembl version 38.73) by using GSNAP [79] with default parameters. Reads for each gene are counted with htseq-count [80]. Finally, raw count matrices for each dataset are used as input in the ZetaSuite pipeline adapted for scRNA-seq analysis to calculate a *ζ* score for each cell with default parameters.

The placenta raw count matrix is downloaded from https://jmlab-gitlab.cruk.cam.ac.uk/publications/EmptyDrops2017-DataFiles. The cell annotation based on CellRanger and EmptyDrops are downloaded from https://github.com/MarioniLab/EmptyDrops2017/tree/master/analysis/placenta. Raw count matrices are used as input for ZetaSuite to calculate a *ζ* score for each cell, and the cutoff is selected based on the *ζ* score distribution and the reflection point for the second cell population (see Fig. 7j). Cells detected by CellRanger, EmptyDrops, and ZetaSuite are analyzed with Seurat [20]: the gene expression matric in each dataset is first normalized with the NormalizeData function and top 2000 features with high cell-to-cell variation are kept for further analysis. The ScaleData function is next used to generate the line-transformation scaled data and the RunPCA function is used to reduce the dimensionality of the dataset. Top 40 principal components are selected according to the ElbowPlot, DimHeatmap, and JackStrawPlot functions. Finally, the FindNeighbors and FindCluster functions are used to cluster cells and the RunUMAP function with default setting is used to perform the nonlinear dimensional reduction.

## Supplementary Information

Additional file 1: Supplementary Figure 1. Overview of in-house data set and the ZetaSuite flowchart. a, In-house data format. Two-dimensional in-house data are generated from a siRNA screen to identify global splicing regulators. In each siRNA-treated well, 407 alternative splicing (AS) events are interrogated by RNA Annealing Selection Ligation sequencing (RASL-seq). A total number of 18,480 siRNA pools against annotated protein-coding genes in the human genome are arrayed in 57 384-well plates. Each plate also contains 6 negative controls (NS-mix), 5 positive controls (siPTBP1) and 5 killer controls (siNEK6). After screening, raw data are tabulated in a matrix as the log_2_ isoform ratio (exon included isoform/exon skipped isoform). b, Flowchart of the ZetaSuite software in three parts (https://github.com/YajingHao/ZetaSuite), as detailed in the text. Supplementary Figure 2. Data analysis using existing statistical approaches. a-b, Z-score distribution of all non-expressors (n=5006) based on 5 randomly selected AS events (a) or all interrogated AS events (b). Red-marked dots indicate hits with Z-score>=3, showing the majority (~80%, see Fig. 2c) of non-expressors scored as false-positive hits when all measured AS events are included in the analysis with the traditional Z-score-based approach. c-d, The Z-score rank distribution (from induced exon skipping on left to exon inclusion on right) of *SF3B1* (c) and *SRSF2* (d) responsive AS events among total detected AS events in the screen, showing skewing of *SF3B1*-induced splicing toward exon skipping and *SRSF2*-induced splicing in both directions. e, Summary of hit numbers at common FDR cutoffs using 4 different existing methods. Supplementary Figure 3. Use of weighted ζ-scores to characterize screen hits. a, List of 10 known splicing regulators displayed in the Zeta plot in main Fig. 3a. b, The density of gene expression levels for annotated core spliceosome genes compared to all other genes in HeLa cells. c, Weighted ζ-scores of representative core spliceosome genes in induced exon skipping (blue) or inclusion (purple), emphasizing that knockdown of core spliceosome components predominately induce exon skipping. d, Comparison of AUPRC among different methods in simulated datasets. Weighted ζ-score in two directions calculated by ZetaSuite are combined in this analysis to reflect the overall functional consequence. This is not applicable to other software, and we thus display the data separately. Supplementary Figure 4. Strategy to remove off-target effects and optimal readouts for two-dimensional genome-wide screens. a, Setting the threshold by using the balanced error level (BRL) approach. Arrow indicates the chosen threshold and associated FDR. b, SS plots for five genome-wide RNAi screens, showing the calculated balance points in each (top panel 1 to 5). One dataset (panel 5) was permutated 5 times to illustrate the lack of a balance point if the data quality is compromised (bottom panel). c-d, Hits with related functions or due to off-target effects. Results of our secondary screen with 4 individual siRNAs in comparison with the pool of those siRNAs (left) or with the pools of other siRNAs against genes that show significant functional similarity (right, reflected by circle size). Hits are due to related functions when multiple single siRNAs produce similar results (c) or to off-target effects when a single siRNA is responsible for the similarity to both siRNA pools (d). e, Deduced SVM curves using two different sets of positive controls. SVM1 is defined with siPTBP1 repeats and SVM2 with a set of known spliceosome components listed in Supplementary Fig. 3a. f, Diagram to illustrate the calculation of weighed ζ-scores without using a SVM curve. g, Venn diagrams showing the overlaps of high confidence hits selected using different SVM curves (left) or with and without using a SVM curve (right). h, Impact of readout (AS event) size on the efficiency in recovering a set of reference hits. Each bar represents the percentage of recovered reference hits (purple for siPTBP1 replicates; green for high confidence hits based on total AS events) by ZetaSuite using different numbers of readouts. Error bars represent the standard deviation from three independent samplings. Supplementary Figure 5. Significantly increased number of fitness genes identified by ZetaSuite from the existing DepMap and DRIVE datasets. a, Comparison of cell lines surveyed by DepMap and DRIVE. Cell lines derived from different cancer types are color-indicated. A common set of 113 cell lines has been analyzed by both projects. b, Comparison of genes interrogated by the two projects. c, Robust segregation of positive (purple: annotated essential genes) and negative (green: non-expressors) controls in DepMap (left) and DRIVE (right). d, Comparison of the performance of different methods in DRIVE and DepMap datasets. e, GO term enrichment for newly identified essential genes by ZetaSuite. f, Function enrichment on KEGG pathways for newly identified essential genes by ZetaSuite. g, Top 10 enriched complexes of newly identified essential genes by ZetaSuite. Complexes are from the CORUM database. h, Density plots of correlation between DEMETER cancer dependency score and AGO2 expression (top) or copy number variation (bottom) for different gene sets according to the color key on right. The overlapped and non-overlapped hits correspond to those displayed in main Fig. 5e. Ten genes uniquely detected by DRIVE are labeled, showing that 8 of 10 are distributed with non-hits. Supplementary Figure 6. Functional analysis of identified hits by ZetaSuite. a-b, Averaged dependency scores of *MYC* (a) and *ATR* (b) in different cancer tissues. c, Association of ZetaSuite-identified cancer dependencies with gene expression, copy number and mutation features as in main Fig. 6e. d, Clusters of hits detected by ZetaSuite that leads to improved tumor cell proliferation. e, Global network of tumor checkpoint hits. Highlighted sub-networks include those involved in the regulation of GTPase activities, DNA packaging, and protein phosphorylation. Supplementary Figure 7. Application of ZetaSuite to single-cell transcriptomics. a, ζ-plot at each bin over a full range of gene expression. The number of expressed genes is based on the benchmark dataset (PRJEB4039). High-quality cells (orange) or low-quality cells (blue) are indicated with the upper panel to compare with annotated empty cells and with lower panel with annotated broken cells. b, Violin plots of the distribution of broken, empty and high-quality cells according to different metrics. c, ROC curves are deduced using different metrics. The p-values are calculated by plot.roc in pROC R package with default parameters. d-e, UMAP of cells identified by CellRanger, EmptyDrops or ζ cut-off. Colors were labeled by T cell marker gene expression(d) or Monocyte marker gene expression (e).

Additional file 2. Review history.

## Data Availability

The datasets used to evaluate the existing and newly designed methods are available from Gene Expression Omnibus with the accession number of GSE207344 [81]. The DRIVE [10] and DepMap [11] data already processed with DEMETER2 are downloaded from https://depmap.org/portal/download/. The raw sequencing reads from two scRNA-seq benchmark datasets are respectively downloaded (E-GEOD-48968 [63] and PRJEB4039 [64]) from ArrayExpress Archive and European Nucleotide Archive. scRNA-seq generated from the placenta is available in ArrayExpress, with experiment codes E-MTAB-6701 [65]. ZetaSuite has been implemented as an R package, which is available on CRAN (https://cran.r-project.org/web/packages/ZetaSuite/index.html). The open source ZetaSuite Perl module and stepwise guide for its usage are freely available from the website https://github.com/YajingHao/ZetaSuite [82]. We will update this website periodically with new versions. All codes, datasets, and a singularity definition file to reproduce the computational environment along with the scripts to reproduce every figure or table can be found at Zenodo under DOI: 10.5281/zenodo.6395174 [83].
