# Supplementary material for: ZetaSuite: computational analysis of two-dimensional high-throughput data from multi-target screens and single-cell transcriptomics
Source: Genome Biol. 2022 Jul 25;23:162. doi: 10.1186/s13059-022-02729-4 (PMC9310463; doi:10.1186/s13059-022-02729-4)
Supplement: Supplementary file 1 — Additional file 1: Supplementary Figure 1. Overview of in-house data set and the ZetaSuite flowchart. a, In-house data format. Two-dimensional in-house data are generated from a siRNA screen to identify global splicing regulators. In each siRNA-treated well, 407 alternative splicing (AS) events are interrogated by RNA Annealing Selection Ligation sequencing (RASL-seq). A total number of 18,480 siRNA pools against annotated protein-coding genes in the human genome are arrayed in 57 384-well plates. Each plate also contains 6 negative controls (NS-mix), 5 positive controls (siPTBP1) and 5 killer controls (siNEK6). After screening, raw data are tabulated in a matrix as the log2 isoform ratio (exon included isoform/exon skipped isoform). b, Flowchart of the ZetaSuite software in three parts (https://github.com/YajingHao/ZetaSuite), as detailed in the text. Supplementary Figure 2. Data analysis using existing statistical approaches. a-b, Z-score distribution of all non-expressors (n=5006) based on 5 randomly selected AS events (a) or all interrogated AS events (b). Red-marked dots indicate hits with Z-score>=3, showing the majority (~80%, see Fig. 2c) of non-expressors scored as false-positive hits when all measured AS events are included in the analysis with the traditional Z-score-based approach. c-d, The Z-score rank distribution (from induced exon skipping on left to exon inclusion on right) of SF3B1 (c) and SRSF2 (d) responsive AS events among total detected AS events in the screen, showing skewing of SF3B1-induced splicing toward exon skipping and SRSF2-induced splicing in both directions. e, Summary of hit numbers at common FDR cutoffs using 4 different existing methods. Supplementary Figure 3. Use of weighted ζ-scores to characterize screen hits. a, List of 10 known splicing regulators displayed in the Zeta plot in main Fig. 3a. b, The density of gene expression levels for annotated core spliceosome genes compared to all other genes in HeLa cells. c, Weighted ζ-s [file 13059_2022_2729_MOESM1_ESM.docx]

**Supplementary Figure 1**

**
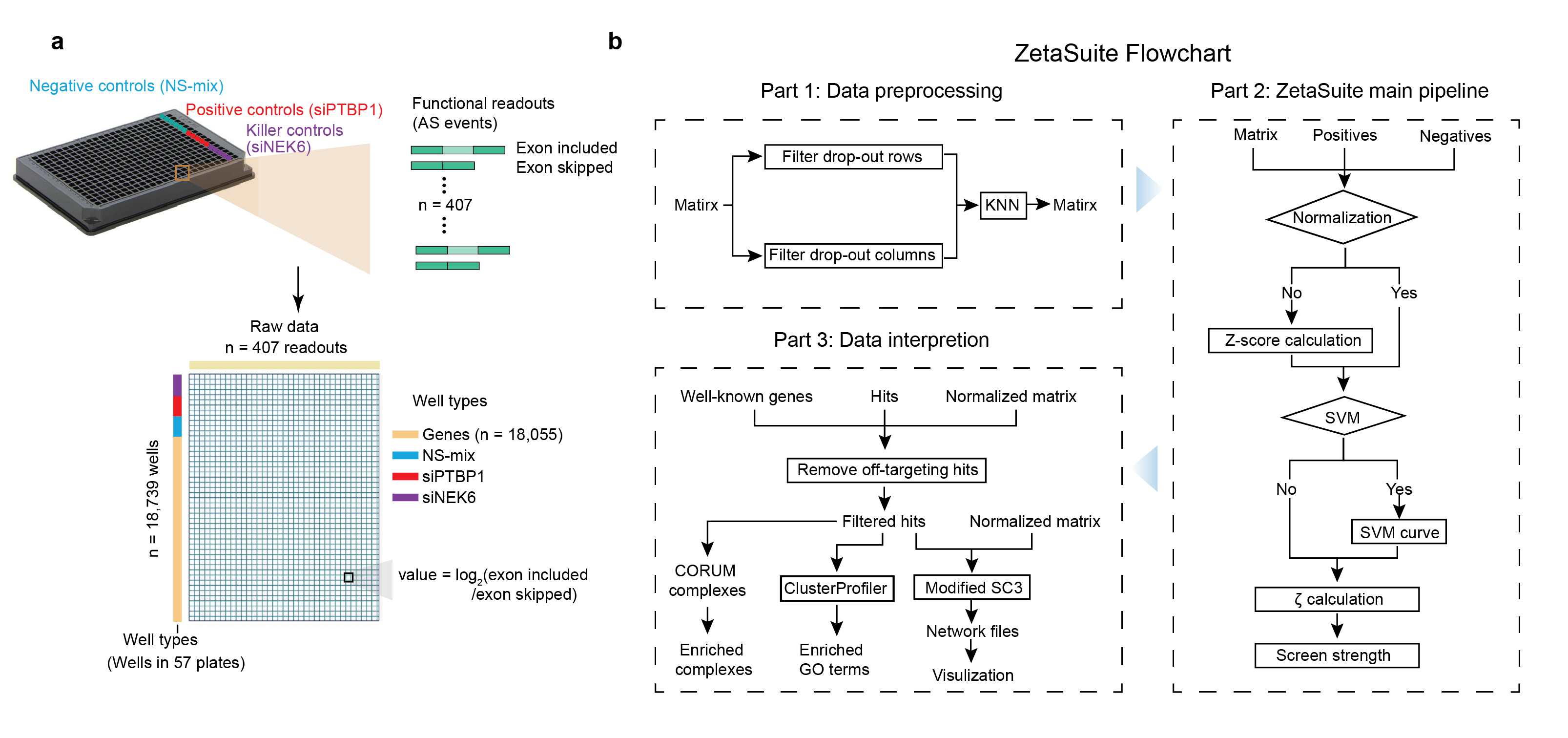
**

**Supplementary Figure 1.** **Overview of in-house data set and the ZetaSuite flowchart.**

**a**, In-house data format. Two-dimensional in-house data are generated from a siRNA screen to identify global splicing regulators. In each siRNA-treated well, 407 alternative splicing (AS) events are interrogated by RNA Annealing Selection Ligation sequencing (RASL-seq). A total number of 18,480 siRNA pools against annotated protein-coding genes in the human genome are arrayed in 57 384-well plates. Each plate also contains 6 negative controls (NS-mix), 5 positive controls (siPTBP1) and 5 killer controls (siNEK6). After screening, raw data are tabulated in a matrix as the log_2_ isoform ratio (exon included isoform/exon skipped isoform). **b**, Flowchart of the ZetaSuite software in three parts (<https://github.com/YajingHao/ZetaSuit>e), as detailed in the text.

**Supplementary Figure 2**


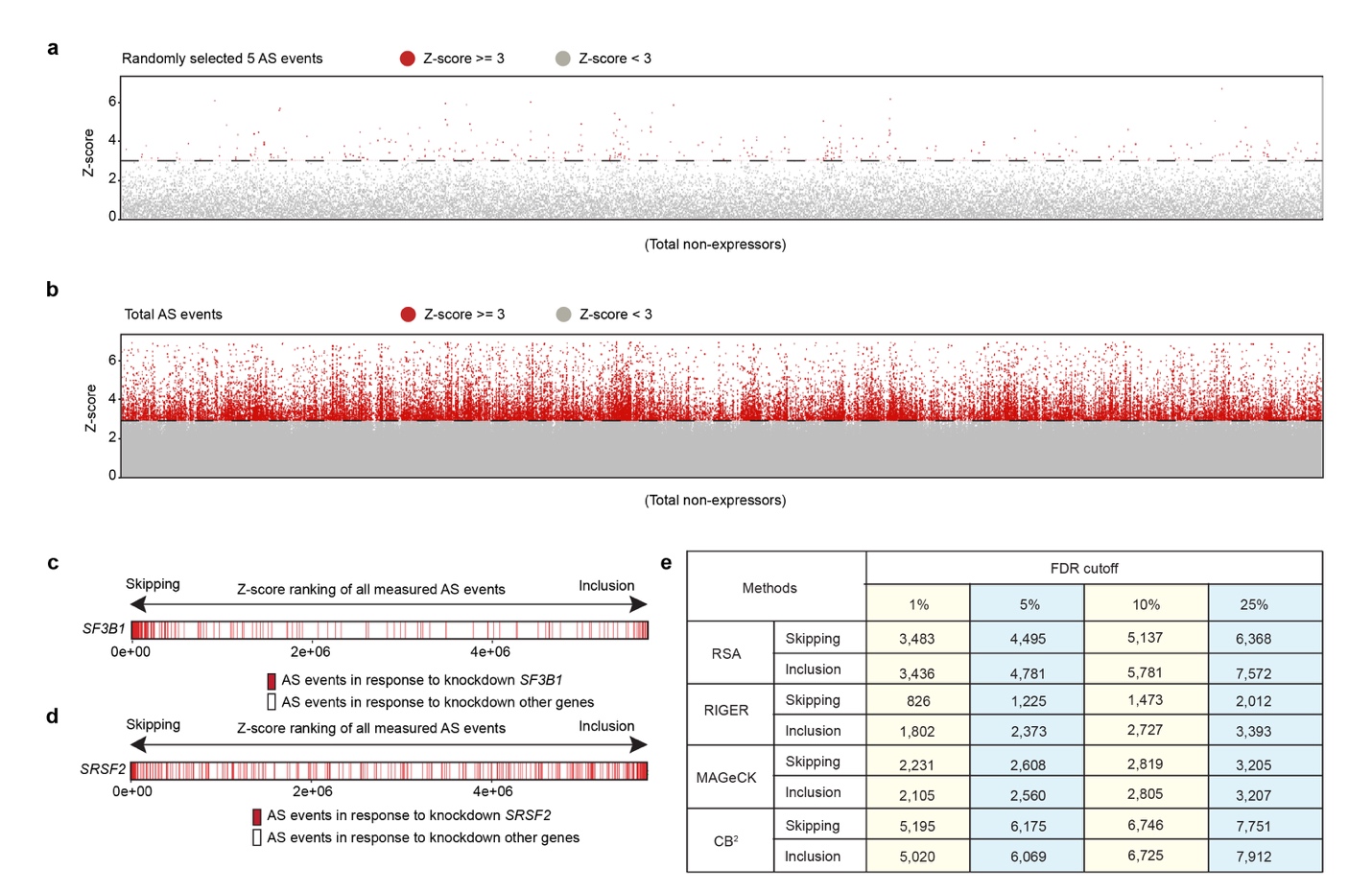


**Supplementary Figure 2. Data analysis using existing statistical approaches.**

**a**-**b**, Z-score distribution of all non-expressors (n=5006) based on 5 randomly selected AS events (**a**) or all interrogated AS events (**b**). Red-marked dots indicate hits with Z-score>=3, showing the majority (~80%, see Fig. 2**c**) of non-expressors scored as false-positive hits when all measured AS events are included in the analysis with the traditional Z-score-based approach. **c**-**d**, The Z-score rank distribution (from induced exon skipping on left to exon inclusion on right) of *SF3B1* (**c**) and *SRSF2* (**d**) responsive AS events among total detected AS events in the screen, showing skewing of *SF3B1*-induced splicing toward exon skipping and *SRSF2*-induced splicing in both directions. **e**, Summary of hit numbers at common FDR cutoffs using 4 different existing methods.

**Supplementary Figure 3**

**
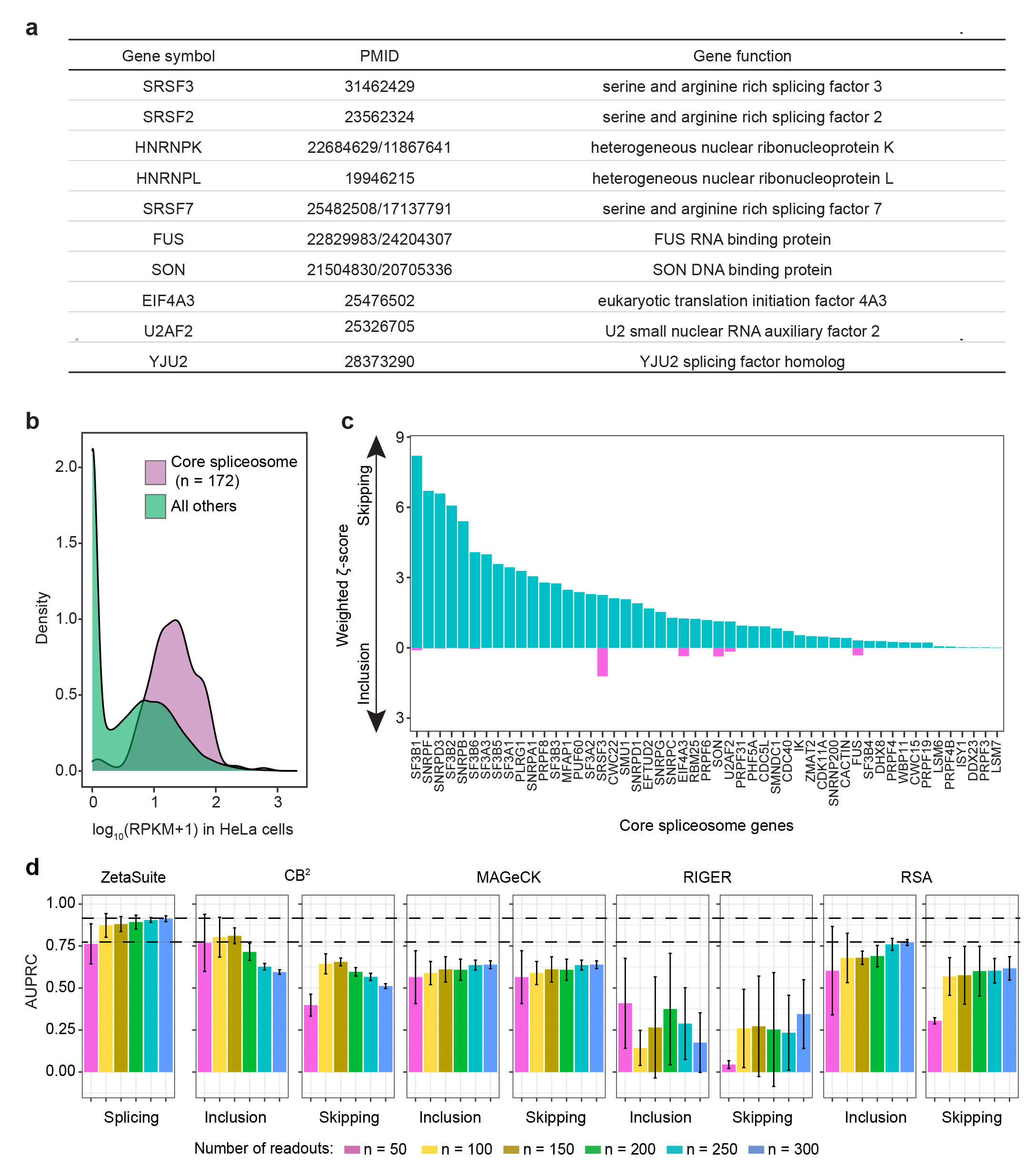
**
**Supplementary Figure 3. Use of weighted ζ-scores to characterize screen hits.**

**a**, List of 10 known splicing regulators displayed in the Zeta plot in main Fig. 3a. **b**, The density of gene expression levels for annotated core spliceosome genes compared to all other genes in HeLa cells. **c**, Weighted ζ-scores of representative core spliceosome genes in induced exon skipping (blue) or inclusion (purple), emphasizing that knockdown of core spliceosome components predominately induce exon skipping. **d**, Comparison of AUPRC among different methods in simulated datasets. Weighted ζ-score in two directions calculated by ZetaSuite are combined in this analysis to reflect the overall functional consequence. This is not applicable to other software, and we thus display the data separately.

**Supplementary Figure 4**
**
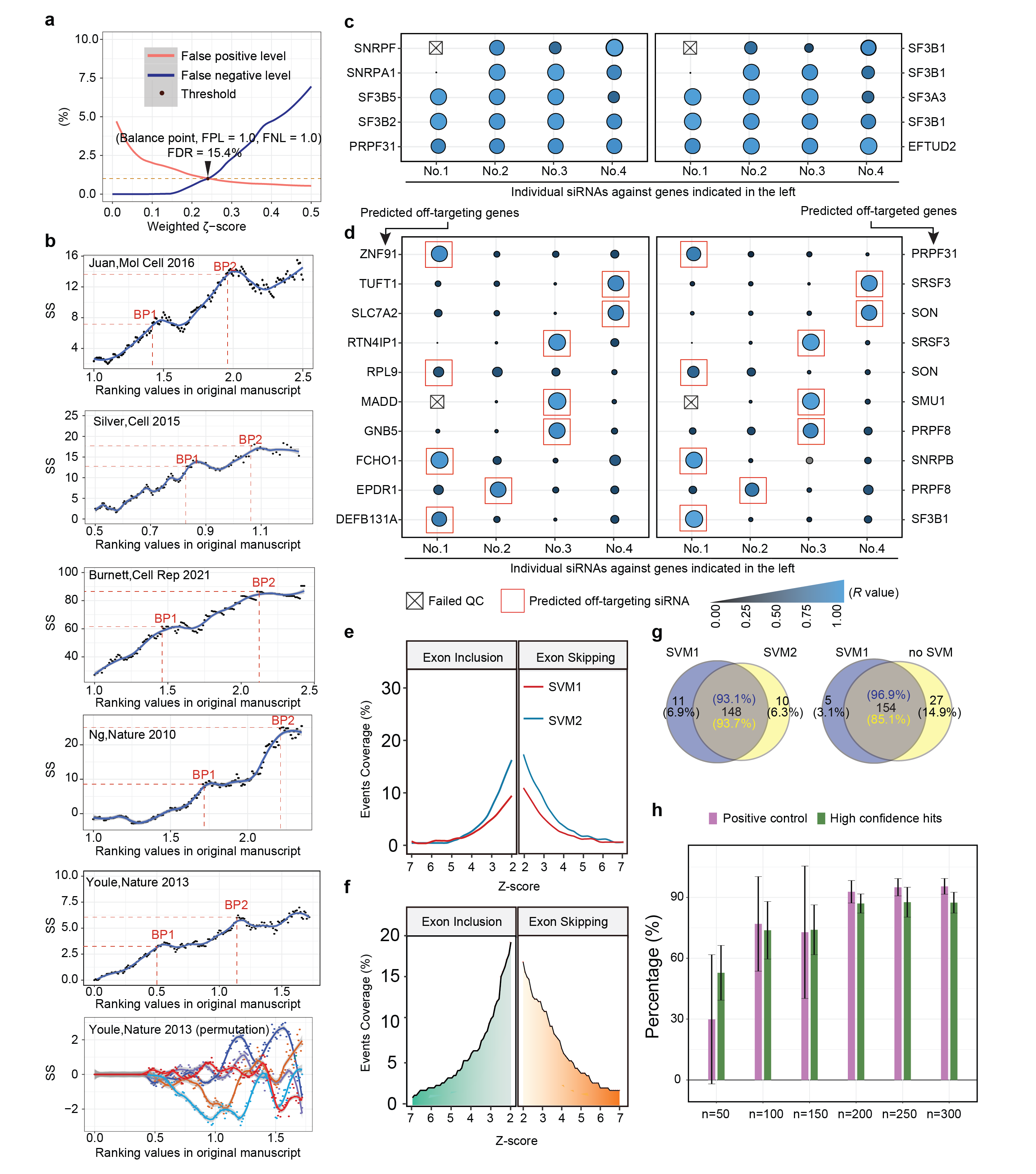
**

**Supplementary Figure 4. Strategy to remove off-target effects and optimal readouts for two-dimensional genome-wide screens.**

**a**, Setting the threshold by using the balanced error level (BRL) approach. Arrow indicates the chosen threshold and associated FDR. **b**, SS plots for five genome-wide RNAi screens, showing the calculated balance points in each (top panel 1 to 5). One dataset (panel 5) was permutated 5 times to illustrate the lack of a balance point if the data quality is compromised (bottom panel). **c**-**d**, Hits with related functions or due to off-target effects. Results of our secondary screen with 4 individual siRNAs in comparison with the pool of those siRNAs (left) or with the pools of other siRNAs against genes that show significant functional similarity (right, reflected by circle size). Hits are due to related functions when multiple single siRNAs produce similar results (**c**) or to off-target effects when a single siRNA is responsible for the similarity to both siRNA pools (**d**). **e**, Deduced SVM curves using two different sets of positive controls. SVM1 is defined with siPTBP1 repeats and SVM2 with a set of known spliceosome components listed in Supplementary Fig. 3a. **f**, Diagram to illustrate the calculation of weighed ζ-scores without using a SVM curve. **g**, Venn diagrams showing the overlaps of high confidence hits selected using different SVM curves (left) or with and without using a SVM curve (right). **h**, Impact of readout (AS event) size on the efficiency in recovering a set of reference hits. Each bar represents the percentage of recovered reference hits (purple for siPTBP1 replicates; green for high confidence hits based on total AS events) by ZetaSuite using different numbers of readouts. ﻿Error bars represent the standard deviation from three independent samplings.

**Supplementary Figure 5**


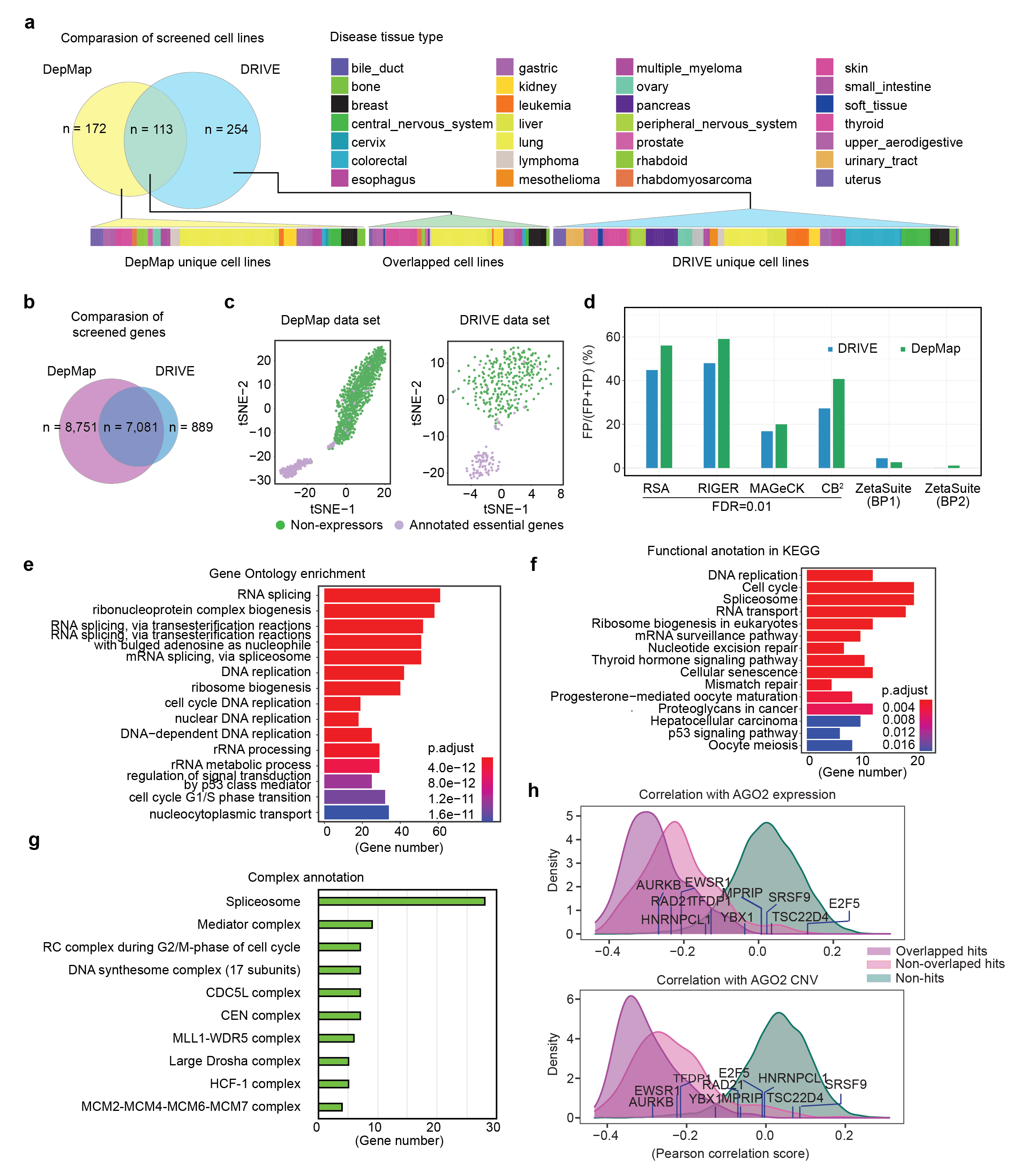


**Supplementary Figure 5.** **Significantly increased number of fitness genes identified by ZetaSuite from the existing DepMap and DRIVE datasets.**

**a**, Comparison of cell lines surveyed by DepMap and DRIVE. Cell lines derived from different cancer types are color-indicated. A common set of 113 cell lines has been analyzed by both projects. **b**, Comparison of genes interrogated by the two projects. **c**, Robust segregation of positive (purple: annotated essential genes) and negative (green: non-expressors) controls in DepMap (left) and DRIVE (right). **d**, Comparison of the performance of different methods in DRIVE and DepMap datasets. **e**, GO term enrichment for newly identified essential genes by ZetaSuite. **f**, Function enrichment on KEGG pathways for newly identified essential genes by ZetaSuite. **g**, Top 10 enriched complexes of newly identified essential genes by ZetaSuite. Complexes are from the CORUM database. **h**, Density plots of correlation between DEMETER cancer dependency score and AGO2 expression (top) or copy number variation (bottom) for different gene sets according to the color key on right. The overlapped and non-overlapped hits correspond to those displayed in main Fig. 5e. Ten genes uniquely detected by DRIVE are labeled, showing that 8 of 10 are distributed with non-hits.

**Supplementary Figure 6**


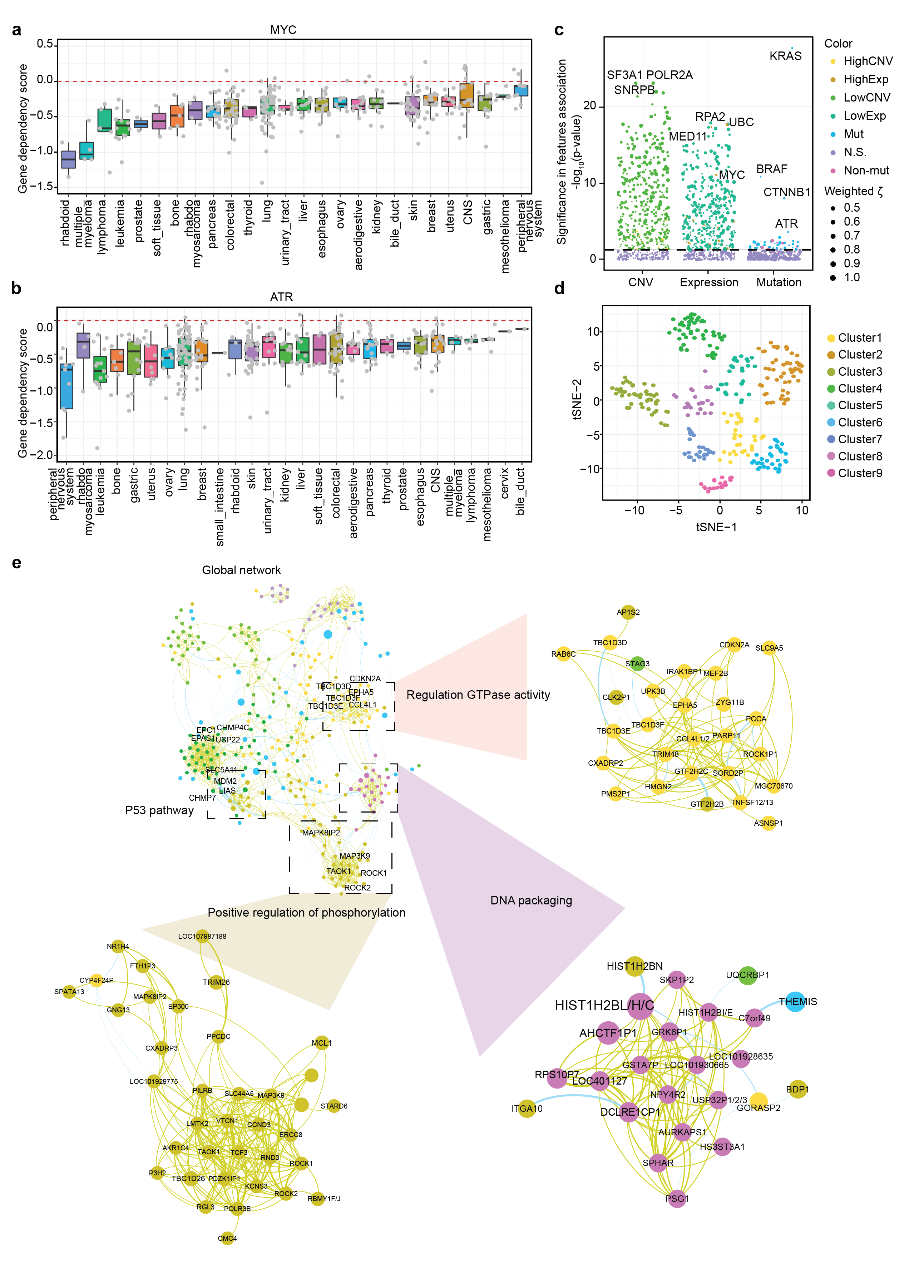


**Supplementary Figure 6. Functional analysis of identified hits by ZetaSuite.**

**a**-**b**, Averaged dependency scores of *MYC* (**a**) and *ATR* (**b**) in different cancer tissues. **c**, Association of ZetaSuite-identified cancer dependencies with gene expression, copy number and mutation features as in main Fig. 6e. **d**, Clusters of hits detected by ZetaSuite that leads to improved tumor cell proliferation. **e**, Global network of tumor checkpoint hits. Highlighted sub-networks include those involved in the regulation of GTPase activities, DNA packaging, and protein phosphorylation.

**Supplementary Figure 7**

**Supplementary Figure 7. Application of ZetaSuite to single-cell transcriptomics**

**a,** ζ-plot at each bin over a full range of gene expression. The number of expressed genes is based on the benchmark dataset (PRJEB4039). High-quality cells (orange) or low-quality cells (blue) are indicated with the upper panel to compare with annotated empty cells and with lower panel with annotated broken cells. **b,** Violin plots of the distribution of broken, empty and high-quality cells according to different metrics. **c,** ROC curves are deduced using different metrics. The p-values are calculated by plot.roc in pROC R package with default parameters. **d-e**, UMAP of cells identified by CellRanger, EmptyDrops or ζ cut-off. Colors were labeled by T cell marker gene expression(d) or Monocyte marker gene expression (e).
