# Supplementary material for: ZetaSuite: computational analysis of two-dimensional high-throughput data from multi-target screens and single-cell transcriptomics
Source: Genome Biol. 2022 Jul 25;23:162. doi: 10.1186/s13059-022-02729-4 (PMC9310463; doi:10.1186/s13059-022-02729-4)
Supplement: Supplementary file 2 — Additional file 2. Review history. [file 13059_2022_2729_MOESM2_ESM.docx]

Review History

**First round of review**

**Reviewer 1**

**Were you able to assess all statistics in the manuscript, including the appropriateness of statistical tests used?** Yes.

**Were you able to directly test the methods?** No.

**Comments to author:**

The authors propose ZetaSuite, a new computational pipeline for analyzing two-dimensional data, with applications to cancer screen datasets and single-cell transcriptomic datasets. The cancer application shows promising potential of this pipeline. I would like to see this paper published after all the following concerns are fully addressed:

Major:
1) In describing the motivation of this work, the authors claim that multiple testing correction methods cannot reduce false positives. They call an siRNA a hit if one of its readout has |Z| > 3, corresponding to a p-value cutoff < 0.01. Then they apply the Bonferroni correction across the siRNAs to correct for multiple testing. This procedure only corrects the error accumulation resulting from multiple siRNA pools; it completely neglects the random error accumulation due to the increasing readout number, which is the main point of their argument. Instead of using |Z| > 3, I suggest authors select a threshold using the Gumbel distribution, which models of the maximum value of n (n is the total number of readout) observations, and then apply multiple test correction procedure.
2) The selection of balance point. The detection of hits is obviously sensitive to the choice of balance point. Some of their applications look at two balance point, and some has zero balance point. I suggest authors give more detailed guidance on how to select a balance point and what to do in cases of no apparent balance point.
3) In the application of cancer cells, the authors performed GO term analysis to unique hits identified by ZetaSuite. I don't understand why identifying key house-keeping genes demonstrated the "effectiveness and objectiveness of ZetaSuite in identifying cancer dependencies".
4) In the single-cell application, authors applied ZetaSuite to QC scRNA-seq data by differentiating damaged cells from high-quality cells. In addition to removing damaged cell, perhaps a more important step is to remove doublets, i.e., two cells that are mistakenly sequenced as one cell. There are multiple methods designed for this purpose, including DoubletFinder. I suggest authors clarify how users should use ZetaSuite to QC scRNA-seq data (e.g., should users use ZetaSuite before applying the doublet detection algorithms), and tailor their subsequent analysis accordingly.

Minor:
1) Typos and grammatical errors: line 67, 'high-quantity cells'; line 174, incomplete sentence; line 322, 'referring' ->'referred'; line 382, 'dare' -> 'are'.
2) In method section "Generation of Zeta plot", Z_max and Z_min are misspecified in line 621 and 622. And from the description, it wasn't clear that the percentage of readouts is computed for each siRNA until I saw figure 3a.
3) In line 638, how is the area calculated if a bin has more than one readout.
4) In figure 1b, the formula of Z-score is inconsistent with that in the methods section. In figure 1c, the y-axis label is missing.

**Reviewer 2**

**Were you able to assess all statistics in the manuscript, including the appropriateness of statistical tests used?** No.

**Were you able to directly test the methods?** No.

**Comments to author:**

Hao et al summarize a pipeline for correcting the amplification of errors in high throughput datasets with multiple readouts. The set of challenges that are unaddressed by other methods that ZetaSuite, the proposed new method, is meant to compensate for are listed across different sections of the results. Though wide in scope it would appear that the authors are addressing two major issues. 1. That "screen specificity is progressively diminished as the number of readouts increases" (line 180) and 2. The challenge to find an optimal cutoff for hits as "a stringent cutoff would miss some valid hits while a loose cutoff would introduce various unwanted artifacts" (68-69). For the first challenge the authors suggest a method that can rank hits in confidence between the applied Z score and SVM learning curve. The addition of ranked confidence values to hits would be a useful contribution to the field of candidate prioritization. For the second challenge the authors suggest targeting known "non-expressors" genes that would presumably provide established true negatives for the screen analysis, however this does not adequately account for off-target effects of these controls against true positive genes. Various other challenges in hit calling are brought up across the manuscript (ie the missing of cells with low nCount - line 74, contamination of low quality cells - line 76), however a more clear and concise summary of the primary challenges the approach seeks to address would increase the overall clarity of the manuscript. While making an admirable attempt to address some of the major issues in multi-readout analysis, the manuscript in its current form would require substantial additional work in validation, writing, and presentation to warrant publication in Genome Biology.

Main points:

1) The manuscript does not address how the approaches adopted in ZetaSuite compare to previous attempts to offer similar solutions. For example, the authors suggest the use of flagging "a significant sequence complementarity to the transcript targeted by another siRNA pool" to identify the off-target effects of RNAi guides, this approach bears striking similarity to the Common Seed Analysis approach suggested by PMID: 22086724 and other solutions like PMID: 22343343 and PMID: 24934636. The authors suggest an approach for finding the inflection point in a list of scores ("balance point" line 239) which would benefit from a discussion of how it compares and differs from the approach previously proposed by PMID: 19564846 which sought to address a similar challenge. The authors proposal of the use of negative controls and non-expressors should be compared and distinguished from methods like PMID: 31015259. There have also been other manuscripts that have attempted to address the challenges of choosing between stringent and lenient cutoffs such as PMID: 33894945. The authors should provide a clear discussion of similar work and how the presented work compares and differs from those that already exist.

2) Another critical issue for the authors to address is how this method is being tested and validated. The work is introduced as having been developed and tested using the alternative splicing and RNAi dataset created by the authors, yet they provide a citation (citation 22, line 89) which is for a manuscript that is "forthcoming" (lines 859-862). In line 287-292 the authors describe an additional test that is applied to different data points within the same dataset. It is not appropriate to present a new method where readers cannot access the same dataset to validate the authors claims.

3) When testing the cancer data set for finding a balance point according to the ZetaSuite method the authors explain "we were unable to derive any balance point with the dataset of DepMap, likely due to scattered data from a relatively smaller number of cell lines surveyed" (lines 329-331). This raises concerns as to the utility of the Zeta Suite method if critical steps in the pipeline to hit selection are likely to fail with data that are not specifically designed for this analysis platform. The authors do mention the use of a benchmark dataset (line 438), a better explanation of which would help the reader assess the rigorousness of the proposed method. Additionally, validating results by pointing to individual publications where that candidate is mentioned in that context is a weak form of validation, as it's always possible to find a listing for different genes in various contexts. Listing a few candidates and showing that they show up in other studies should not take the place of rigorous testing by benchmarked or gold standard datasets. The authors should demonstrate a clear and rigorous testing of the proposed method that doesn't rely so heavily on their in-house dataset. This testing should inform the design and presentation of the method.

4) Without further clarification, it would appear that the particular amalgam of data challenges presented in the dataset are unique to the specific datasets that the authors were using in the preparation of a parallel manuscript. The described pipeline appears to rely on the availability of a specific type of dataset with certain classes of controls and targeting of known non-expresser genes. It is unclear how this method can be used when not all these criteria are met and it seems unlikely that many studies would find themselves with this particular set of data types, sizes, and a priori known expression profiles. The authors should present a clear demonstration of how these challenges might show up as bottlenecks in the use of their method and various ways these issues could be addressed by potential users of the ZetaSuite platform.

5) A final critical omission from this manuscript is a clear guide for how a user can apply this method to their data. As a method, the manuscript does not include a guide to the data types and steps that it would take for readers to use or consider whether ZetaSuite is applicable to their data.


Minor points:
1) The authors make frequent dispositive statements without providing proper citations, such as line 171 ("well-known splicing regulator PTB"), line 195 ("In certain applications, it may be necessary.." which applications?), lines 221-22 ("FPL will gradually decrease while FNL will progressively increase."). The last point in particular. The authors claim that with more readouts the noise would increase, that is counter -intuitive (it is not often that the noise of one data set resembles the noise of one generated by a different assay) the authors should demonstrate that is the case in more than one data set.

2) There are many grammatical errors, typos and omissions throughout the manuscript which would benefit from careful editing. Examples include "Hit selection based on reflection points in the Screen Strength plot" (line 217), and "Any screen requires a cutoff to maximize true positives while minimize true negatives." (line 218), presumably you would want to maximize true negatives and minimize false negatives.

**Reviewer 3**

**Were you able to assess all statistics in the manuscript, including the appropriateness of statistical tests used?** No.

**Were you able to directly test the methods?** No.

**Comments to author:**

1. Your paper does not provide proper account (either theoretical derivations or results from simulation studies) of the statistical operating characteristics of your proposed approach including a comparisons to the statistical operating characteristics to other methods. How does a use assess the strength of the evidence in the results. You have a decision rule (eg. the Strength Screen). What are its statistical properties (e.g., bias, error control)

2. You state "We herein introduce ZetaSuite, a statistical package initially designed to score hits from two-dimensional RNAi screens." This is very confusing. I assume that ZetaSuite is new package.

3. Your github page hosts a large collection of mostly undocumented R and bash scripts. Given that you present this paper as software paper "ZetaSuite, a statistical package" you should consider producing a fully documented package. Looking at your wiki page, most of
the quantitative work was implemented in R so it would make sense to publish an R package to Bioconductor or CRAN. The example on the wiki page can serve as an initial version of the package vignette. Moreover you should provide a singularity or Docker definition file to reproduce the computational environment along with the scripts to reproduce every figure or table in your paper using this package

4. Reading your paper, I was confused with your references to statistics, software package, methods and metrics.
For example L70: "Three methods were commonly used for such quality control (QC) purpose". I assume that you are referring to the three metrics (not methods) as you only list Seurat and Emptydrops (two). Also Seurat is not a method. It is a software package. I assume that zeta "score" (L631) is the zeta statistic. Buit you also use the term "score" for the Z transformation

5. You state that L78: "In this study, we introduce a new zeta statistic by taking two critical QC metrics into consideration without relying on the selection of an arbitral cutoff." This gives the impression that there is an _existing_ class of statistics called zeta statistics and that you are introducing a new variant (this would be akin to saying we are developed a new score statistic or a new likelihood ratio statistic). But aside from that, I have concerns that you are not getting around arbitrary cutoffs. For example, you are using an aribitrary
cutoff to declare an isoform detectable (L588: "in our study, a value is missing if one of the mRNA isoforms is undetectable") . You are using a bin size of 100. Your method will likely be sensitive to the choices of these tuning parameters that you have picked in an arbitrary fashion without let's say cross-validation or using independent data sets.

6. Why is the zeta score in L635 subscripted by i (the left hand side)?

7. What is meant with |Z| < 2 implies P-value is > 0.05. What is Z? Is the Z score or Z statistics (again you use both the terms statistic L604 and score and L606) or does or denote a standard normal random variable? What is the sampling distribution? is |Z| < 2 a rejection region or is |Z|>2 a rejection region? This is another example where your statistical arguments are vague and hard to follow

Editorial comments

1. Please carefully proof read your submission. Starting with the first sentence of your paper:

"Genome-wide screen by RNA interference (with siRNA or shRNA)[1-3] or CRISPR/Cas44 (with sgRNA) has become powerful tools for functional genomics studies."
Other examples
L70: arbitral (should be arbitrary)
L76: expanse (should be expense)

2. Not all readers are familiar with the jargon used in your paper (e.g., cancer dependency). The reader should not have to read the provided reference just to get a basic idea what the jargon is referring to

3. Consider adding an outline of your paper to the background section to orient readers

**Authors Response**

**Point-to-point response to reviewers**

**Reviewer #1:**

*The authors propose ZetaSuite, a new computational pipeline for analyzing two-dimensional data, with applications to cancer screen datasets and single-cell transcriptomic datasets.* *The cancer application shows promising potential of this pipeline. I would like to see this paper published after all the following concerns are fully addressed:*

*Major:*

*1) In describing the motivation of this work, the authors claim that multiple testing correction methods cannot reduce false positives. They call an siRNA a hit if one of its readout has |Z| > 3, corresponding to a p-value cutoff < 0.01. Then they apply the* *Bonferroni correction across the siRNAs to correct for multiple testing. This procedure only corrects the error accumulation resulting from multiple siRNA pools; it completely* *neglects* *the random error accumulation due to the increasing readout number, which is the main point of their argument. Instead of using |Z| > 3, I suggest authors select a threshold using the* *Gumbel distribution, which models of the maximum value of n (n is the total number of readout) observations, and then apply multiple test correction procedure.*

Thanks for the suggestion, and accordingly, we have performed the following steps:

1) Extract the maximum |Z-score| value of each siRNA pool; 2) Use R package evd according to the maximum values to estimate the parameters of Gumbel distribution and show that the maximum |Z-score| values indeed exhibit the Gumbel distribution (Fig.R1); and 3) Perform Bonferroni/FDR correction for all p-values in the Gumbel distribution.

We found that the threshold was |Z_score|=18.442 if the Bonferroni corrected p-value was set to be 0.01 (p.adjust=0.01). This led to the selection of only 45 genes, including 7 non-expressed genes, missing all positive controls (Table R1). If p.adjust was loosened to 0.05, the number of hits increased to 61 (50 genes + 11 non-expressors) but still no positive controls were selected. Next, we changed the Bonferroni correction to a more lenient correction, FDR correction, and found that the resulting FDR was still very high (39.3%), even with a lenient cut-off q=0.05.

Figure R1. The parameter estimation of Gumbel distribution.

From these analyses, it appears that any threshold selected from the Gumbel distribution would miss most positive controls, yet with alarmingly high FDR values, which likely resulted from the dominance of random noises that are of high |Z-score| values. We have described these points in the Method section of the revised manuscript.

Table R1. The statistical features of different thresholds.

| Threshold | p.adjust = 0.01 | p.adjust = 0.05 | q = 0.01 | q = 0.05 |
| --- | --- | --- | --- | --- |
| Genes | 38 | 50 | 104 | 197 |
| Non-expressors | 7 | 11 | 37 | 69 |
| Positive controls | 0 | 0 | 2 | 107 |
| FDR | 100% | 100% | 94.9% | 39.2% |
| False positive level | 0.14% | 0.22% | 0.74% | 1.4% |
| False negative level | 100% | 100% | 99.3% | 64.2% |

*2) The selection of balance point. The detection of hits is obviously sensitive to the choice of balance point. Some of their applications look at two balance point, and some has zero balance point. I suggest authors give more detailed guidance on how to select a balance point and what to do in cases of no apparent balance point.*

In a SS plot, the SS value would be progressively elevated (indicating progressive reduction of false positive hits) with increasing cut-off stringency. For example, we extracted 5 public RNAi screening datasets and used non-expressed genes as internal negative controls [1-5]. Interestingly, each of these high-quality screening datasets exhibits two apparent balance points (Fig.R2a-e). Next, we permutated the ranking values of a representative genome-wide screening (from the dataset in Fig.R2e) five times, after which we no longer detected any balance point in the SS plot (Figure R2f). These data are now incorporated as Fig.S4b in the revised manuscript.

Figure R2. SS plots for five genome-wide RNAi screens, showing the calculated balance points in each (a to e). One dataset (from e) was permutated 5 times to illustrate the lack of a balance point if the data quality is compromised (f).

The above analysis suggests that it is rare to lack a balance point with successful screening datasets, and interestingly, the presence of at least one balance point in the SS plot appears to provide a measure of data quality. The biological meaning of one or more balance points may differ with different experiments; from analysis of our own screening data, we identify both direct and indirect splicing regulators based on the first balance point and mostly direct splicing regulators based on the second balance point. The Reviewer may also wonder about a hypothetical situation where increasing stringency leads to progressive increase in the positive rate without a detectable plateau. This would be indicative of the total absence of false positives (off-targets or other non-specific hits), which is practically impossible.

We have now incorporated this information in the revised manuscript to provide a general guidance to future users to select cut-offs based on the SS plot. We state that the presence of at least one balance point is indicative of a successful screening dataset based on our analysis of multiple genome-wide screening datasets. Once a SS plot is generated, changes in SS values are usually visible, which can be identified to aid in the selection of one or two cut-offs according to the error tolerance in a given screening dataset.

*3) In the application of cancer cells, the authors performed GO term analysis to unique hits identified by ZetaSuite. I don't understand why identifying key house-keeping genes demonstrated the "**effectiveness and objectiveness of ZetaSuite in identifying cancer dependencies".*

In fact, this raises a general problem with the term cancer dependency. Functionally speaking, cancer dependency means a gene whose depletion compromises cancer cell growth. The problem here is that most people assume that such cancer dependency is specific to cancer cells, not normal cells. In practice, however, cancer dependency screening is only conducted on a large set of cancer cells without normal cells as controls. As a result, many essential house-keeping genes, such as those involved in transcription, splicing, and translation, are also identified as part of a cancer dependency cohort. Those house-keeping genes are thus indicative of a successful screening dataset, even though they are not further pursued in most studies as cancer dependencies that likely play specific roles in cancer cells, but not normal cells. We similarly use this concept to evaluate the "effectiveness and objectiveness of ZetaSuite in identifying cancer dependencies".

*4) In the single-cell application, authors applied ZetaSuite to QC scRNA-seq data by differentiating damaged cells from high-quality cells. In addition to removing damaged cell, perhaps a more important step is to remove doublets, i.e., two cells that are mistakenly sequenced as one cell. There are multiple methods designed for this purpose, including* *DoubletFinder. I suggest authors clarify how users should use ZetaSuite to QC scRNA-seq data (e.g., should users use ZetaSuite before applying the doublet detection algorithms), and* *tailor their subsequent analysis accordingly.*

Thanks for the valuable suggestion. The main goal of ZetaSuite design is to deal with empty and broken cells, and the contamination of these low quality cells would compromised the following analysis in scRNAseq as demonstrated in our study and other publications[6-8]. We have now compared ZetaSuite with 8 methods designed to remove doublets, including DoubletFinder, by using a simulated dataset generated by scDesign from 80 scRNAseq datasets with varying doublet rates (i.e., different percentages of doublets), sequencing depths, cell types, and between-cell-type heterogeneity levels [9]. As illustrated in Fig.R3, we found that the performance of ZetaSuite (with average AUPRC is 0.75 and average AUROC is 0.8) is above the average of the existing 8 methods specifically designed for doublets elimination, but clearly is not as effective as DoubletFinder under different stimulated conditions. Based on this analysis, if the users require a high precision for removing doublets, we recommend DoubletFinder. Otherwise, ZetaSuite could be a comprehensive tool to eliminate empty droplets, broken cells, and doublets. Because of the space constraint, we have chosen to describe our observation and recommendations without displaying the data on the simulated data in the revised manuscript.

Figure R3. Performance of ZetaSuite relative to 8 published doublet detection methods in four simulation settings: various doublet rates, sequencing depth (from 500 to 10,000 UMI counts per cell, with a step size of 500 counts), number of cell types (from 2 to 20 with a step size of 1) and heterogeneity levels (specify the extent to which genes are differentiated between two cell types).

*Minor:*

*1) Typos and grammatical errors: line 67, 'high-**quantity cells'; line 174, incomplete sentence; line 322, 'referring' ->'**referred'; line 382, 'dare' -> 'are'.*

Corrected.

*2) In method section "Generation of Zeta plot", Z_max and Z_min are misspecified in line 621 and 622. And from the description, it wasn't clear that the percentage of readouts is computed for each siRNA until I saw figure 3a.*

We have corrected the specifications for Z_max and Z_min in the Method section. We have also improved the description: “Next, for each siRNA pool, the percentage of readouts scored above the Z-score cutoff in each bin is determined.”

*3) In line 638, how is the area calculated if a bin has more than one readout.*

Here, we further clarify how we calculate the area at each bin in the Method section of the revised manuscript: We first divide the Z-score range into 100 bins, and at each bin, we determine the number of readouts that show significant changes above the Z-score cutoff at the bin for each siRNA-targeted gene and then divide this number with the total of measured readouts. After subtracting the background percentage (based on the SVM curve), we obtain the increased percentage of readouts that show significant changes. To highlight hits scored at higher Z-score bins, the area in each bin is multiplied with the value of Z-score in such bin and all adjusted areas are summed to give rise to the final weighted ζ score.

*4) In figure 1b, the formula of Z-score is inconsistent with that in the methods section. In figure 1c, the y-axis label is missing.*

Corrected.

**Reviewer #2:**

*Hao et al summarize a pipeline for correcting the amplification of errors in high throughput datasets with multiple readouts. The set of challenges that are unaddressed by other methods that ZetaSuite, the proposed new method, is meant to compensate for are listed across different sections of the results. Though wide in scope it would appear that the authors are addressing two major issues. 1. That "screen specificity is progressively diminished as the number of readouts increases" (line 180) and 2. The challenge to find an optimal cutoff for hits as "a stringent cutoff would miss some valid hits while a loose cutoff would introduce various unwanted artifacts" (68-69). For the first challenge the authors suggest a method that can rank hits in confidence between the applied Z score and SVM learning curve.* *The addition of ranked confidence values to hits would be a useful contribution to the field of candidate prioritization. For the second* *challenge the authors suggest targeting known "non-expressors" genes that would presumably provide established true negatives for the screen analysis, however this does not adequately account for off-target effects of these controls against true positive genes. Various other challenges in hit calling are brought up across the manuscript (ie the missing of cells with low nCount - line 74, contamination of low quality cells - line 76), however a more clear and concise summary of the primary challenges the approach seeks to address would increase the overall clarity of the manuscript.* *While making an admirable attempt to address some of the major issues in multi-readout analysis, the manuscript in its current form would require substantial additional work in validation, writing, and presentation to warrant publication in Genome Biology.*

We appreciate these thoughtful suggestions and have accordingly revised our manuscript as detailed below.

*Main points:*

*1) The manuscript does not address how the approaches adopted in ZetaSuite compare to previous attempts to offer similar solutions. For example, the authors suggest the use of flagging "a significant sequence complementarity to the transcript targeted by another siRNA pool" to identify the off-target effects of RNAi guides, this approach bears striking similarity to the Common Seed Analysis approach suggested by PMID:* *22086724 and other solutions like* *PMID: 22343343 and PMID: 24934636. The authors suggest an approach for finding the inflection point in a list of scores ("balance point" line 239) which would benefit from a discussion of how it compares and differs from the approach previously proposed by* *PMID: 19564846* *which sought to address a similar challenge.* *The authors proposal of the use of negative controls and non-expressors should be compared and distinguished from methods like* *PMID: 31015259. There have also been other manuscripts that have attempted to address the challenges of choosing between stringent and lenient cutoffs such as* *PMID: 33894945. The authors should provide a clear discussion of similar work and how the presented work compares and differs from those that already exist.*

The Reviewer suggests comparing our approach with a series of published methods designed to address related challenges in high throughput screenings. Specifically,

1. Comparing strategies for removing potential off-targets.

The Reviewer first recommended comparison with common seed analysis (PMID: 22086724). This method suggests that siRNA might function as a miRNA on off-targets, and thus, recommends using matches to seed sequences to deduce potential off-targets. Interestingly, all siRNAs scored in our primary screening passed the threshold suggested in this approach (see the attached common seed analysis output in Table R2.csv), yet we still identified many true off-targeting siRNAs based on our secondary screening. It is likely because common seeds are insufficient to identify all off-targeting effects in screening, as emphasized in GESS[10], which is a known fact in the miRNA field.

GESS (PMID: 22343343 and PMID: 24934636) is a bioinformatics method to help design a siRNA library by removing genes that tend to be off-targeted by various siRNAs in the library. The algorithm requires a scorable phenotype associated with a specific gene and asks whether many siRNAs designed to target other genes show miRNA-like seeds on the gene, thereby causing a similar phenotype. This algorithm is thus designed to aid in siRNA library design, which requires a set of defined phenotypes, not for filtering out specific siRNAs for their off-targeting effects in different applications of an existing siRNA library.

In fact, ZetaSuite takes advantage of a similar concept of phenotypic association as in GESS, but without requiring specific match in seed regions. As described in our manuscript, the induction of similar changes in multiple readouts may be considered as a “common” phenotype, and thus, if two siRNAs designed to target two separate genes induce a similar phenotype, ZetaSuite will determine whether the two genes likely function in the same regulatory pathways or one gene may be a likely on-target and the other a potential off-target. To verify our methods in identifying off-targeting genes in primary screening, we deconvoluted the siRNA pools of predicted off-targeting genes into individual siRNAs in secondary screening to identify likely off-targeting siRNAs. The results demonstrated the high confidence of our approach as illustrated in Fig.S4c-d. We have further emphasized this advantage of ZetaSuite in the revised manuscript.

2. Comparing strategies for choosing appropriate cut-off.

In our manuscript, we introduce a concept of “Screen Strength” to identify “balance points”, which helps maximize the number of hits and minimize the apparent false-discovery rate. The Reviewer mentioned another method RNAiCut (PMID:19564846) designed to address a similar challenge. The method is based on the hypothesis that true positive hits identified from an RNAi screening are also densely interconnected in an existing PPI network, which was largely based on RNAi screening performed in *Drosophila*. The problem is that the approach heavily depends on the accuracy of established PPI networks, which is challenging in mammalian cells, and that the recommended selection of minimum p-value as cut-off is not always true, especially for some specific functions that need incorporation of multiple pathways. The major aim of RNAiCut is to find a threshold to minimize false positive hits by coupling the orthogonal PPI network information. In contrast, SS-based approach directly focuses on the false discovery rate according to non-expressed genes, which serves to provide additional information independent of ranking values (ζ scores) of individual hits, and does not depend on any prior functional knowledge. Because the RNAiCut webserver is no longer working, indicating a lack of motivation for the original authors to make continuous efforts in updating the method, we have decided to briefly mention our comparison with RNAiCut in the revised manuscript.

SIGNAL (PMID: 33894945) is another method similar to RNAiCut. The major aim of this software is to prioritize hits based on user-selected hits, requires defining high-confidence and median confidence hits, and then using such defined hits to iteratively perform pathway and network enrichment. A new set of hits is a collection of hits in overlapped enriched pathways and networks. A major drawback of SIGNAL is that novel genes not annotated in known pathways and networks would be missed, thus limiting their discovery. In any case, our SS plot suggests a general strategy to select high and median confidence hits, which can be coupled with SIGNAL. We have now pointed this out in our manuscript.

3. Comparing strategies for rank ordering hits from multi-target screening.

In our manuscript, we compared Zeta statistics with other methods, including Z statistics, SSMD, MAGeCK, RSA, RIGER to rank order hits from high-throughput screening datasets. Following the Reviewer’s suggestion, we have further compared our strategy with the method called CB^2^ (PMID: 31015259). This method was designed to process datasets with multiple sgRNAs against each gene and tested in multiple replicates, thus enabling the Fisher’s combined probability test to combine p-values of individual sgRNAs for a given targeted gene after comparing their differences with a modified student’s t-test. Although this type of datasets is not very common in genome-wide screening, we have nevertheless compared the performance of CB^2^ relative to our Zeta metric. Using our built-in positive controls and internal negative controls, we found that CB^2^ is not suitable to combine multiple readouts to assess the overall strength of individual siRNA pools when the number of readouts is exceedingly large. The new data have been implemented in the current Fig.2h, Fig.S2e and Fig.3e-g. After this, we feel that our current comparison is sufficient to demonstrate the performance of the newly developed Zeta statistics in rank ordering hits from multi-target screening.

In conclusion, we have amended our manuscripts to give credits to previous efforts in addressing critical challenges. Importantly, such systematic comparison has helped us to emphasize many advantages of ZetaSuite in addressing those challenges.

*2) Another critical issue for the authors to address is* *how this method is being tested and validated. The work is introduced as having been developed and tested using the alternative splicing and RNAi dataset created by the authors, yet they provide a citation (citation 22, line 89) which is for a manuscript that is "forthcoming" (lines 859-862).* *In line 287-292 the authors describe an additional test that is applied to different data points within the same dataset.* *It is not appropriate to present a new method where readers cannot access the same dataset to validate the authors claims.*

This is a valid criticism, as we have been struggling in presenting intercalating information in papers that also need to stand alone. The ZetaSuite was initially developed to process our multi-target screening data to identify global splicing regulators. As the data and discoveries become progressively enlarged, we are forced to seek separate publication of development of Zeta statistics for data analysis and resulting biological findings. As a result, we have further expanded the content of our manuscript on ZetaSuite to demonstrate its broadly utility in processing multi-dimensional datasets.

Here, to address the concern of the Reviewer on how our approach is validated, we wish to emphasize that we have used built-in negative (NS-mix) and positive controls (si-PTBP1) to deduce the SVM curves used for calculating the Zeta scores, and then took advantage of non-expressed genes as an independent dataset to validate the performance of Zeta statistics. As ZetaSuite is designed to deal with random error accumulation due to increasing readout number, line 287-292 is meant to describe our efforts in validating the robustness of the ZetaSuite based on different numbers of readouts after simulating the number of readouts. The results showed that ZetaSuite achieves better performance when the number of functional readouts number is increased to >200. Besides using our in-house datasets, we also validated our approach according to benchmarks in cancer dependency datasets and single-cell RNAseq (detailed in Point 3).

The datasets used in the development of the Zeta statistics have been provided at <http://fugenome.ucsd.edu/RASL-seq/> in the “Availability of data and materials” section of our manuscript. To avoid the impression for presenting the data in a “forthcoming” paper, we have removed the citation and directly provided the dataset in Zenodo with DOI: 10.5281/zenodo.6395174.

*3) When testing the cancer data set for finding a balance point according to the ZetaSuite method the authors explain "we were unable to derive any balance point with the dataset of DepMap, likely due to scattered data from a relatively smaller number of cell lines surveyed" (lines 329-331).* *This raise concerns as to the utility of the ZetaSuite method if critical steps in the pipeline to hit selection are likely to fail with data that are not specifically designed for this analysis platform.*

As we responded to a related question of Reviewer 1, point 2, we found that the presence of at least one balance point in the SS plot provides a measure of data quality from a given screening, because the principle of our screen strength (SS) plot is based on the progressive improvement in removing false positives hits with increasing stringency cut-off. The absence of a visible balance point in the SS plot of the DepMap data is similar to that with our permutated data (see Fig.R2f above), suggesting that the lack of balance point reflects low data quality from the DepMap project in “Cancer checkpoint” direction, rather than any utility issues with ZetaSuite. This is further supported by the presence of clear balance points with the DRIVE dataset, which was generated by the same cancer dependency assay as DepMap.

*The authors do mention the use of a benchmark dataset (line 438), a better explanation of which would help the reader assess the* *rigorousness of the proposed method. Additionally,* *validating results by pointing to individual publications where that candidate is mentioned in that context is a weak form of validation, as it's always possible to find a listing for different genes in various contexts. The authors should demonstrate a clear and rigorous testing of the proposed method that doesn't* *rely so heavily on their in-house dataset.* *This testing should inform the design and presentation of the method.*

ZetaSuite has two major applications: one is to define hits in two-dimensional high-throughput screening datasets and the other is to filter low-quality cells in single-cell sequencing datasets.

The Reviewer questioned the utility of ZetaSuite for filtering out low-quality cells in scRNA-seq datasets. In this analysis, we used a benchmark dataset in which individual sequenced cells were visually inspected by microscopy to determine the cell quality in each droplet. We believe that this is the most rigorously quality control we have ever seen with any published scRNAseq datasets in the literature.

Please note that we have used a variety of independent datasets to validate our approach in our in-house HTS^2^ datasets. To further test ZetaSuite in other large-scale screening, we extended ZetaSuite to analysis of two cancer dependency datasets (DRIVE and DepMap). To this end, we divided the cancer dependency datasets into two parts to reflect either cancer dependencies or cancer checkpoints. In the cancer dependency map, “essential genes” and “non-expressors” are provided as benchmarks. In the previous version of our manuscript, we demonstrated that ZetaSuite is able to identify almost all “essential genes” but exclude most “non-expressors”. Now, we have used to the same datasets to compare the performance of ZetaSuite with other packages, including RSA, RIGER, MAGeCK, and CB^2^. We found that ZetaSuite shows the best performance (Fig.R4). We have incorporated this data in revised Fig.S5d.

Figure R4 Comparison of the performance of different methods in DRIVE and DepMap datasets.

The Reviewer seems to question the validation using newly identified genes that are consistent with their documented roles in cancer biology. We wish to emphasize that we present the information to illustrate the capacity of ZetaSuite in making discoveries that make sense. This is NOT the way we tested the utility and ability of ZetaSuite.

In summary, we would like to further stress the fact that, besides using our own screening datasets with built-in positive and negative controls, we used two cancer dependency datasets and two most rigorously and independently characterized scRNA-seq datasets to demonstrate the capacity of ZetaSuite in identifying hits and excluding the empty and broken droplets, respectively.

*4) Without further clarification, it would appear that the particular* *amalgam of data challenges presented in the dataset are unique to the* *specific datasets that the authors were using in the preparation of a parallel manuscript. The described pipeline appears to rely on the availability of a specific type of dataset with* *certain classes of controls and targeting of known non-expresser genes. It is unclear how this method can be used when not all these criteria are met and it seems unlikely that many studies would find themselves with this particular set of data types, sizes, and* *a priori known expression profiles. The authors should present* *a clear demonstration of how these challenges might show up as bottlenecks in the use of their method and* *various ways these issues could be addressed by potential users of the* *ZetaSuite platform.*

The development of the Zeta statistics was initially motived to process our in-house multi-target screening data. We then realize the broad utility of our approach in treating any data with two-dimensional data, i.e. many genes vs many readouts (in contrast to many genes on a single or a few readouts), as we have illustrated with cancer dependency screening and scRNA-seq data besides our own in-house RNAi screening data. We believe that this type of data with two-dimensional matrix will become increasingly common with the advance of high throughput technologies.

The nutshell of the problem is how to treat data with two-dimensional matrix, and we offer a “two-dimensional” solution to the problem by calculating the area under the screening results, rather than a single “one-dimensional” cut-off like the Z statistics. The only requirement for the Zeta statistics is multi-target readouts and the ability to score these readouts with increasing stringency.

In the revised manuscript, we also provide guide to situations lacking certain classes of controls (built-in negative and positive controls) and missing prior expression profiles to deduce non-expressed genes. For lacking built-in controls, we have demonstrated in Fig.S4f-g where even without positive controls to generate SVM curves, ZetaSuite is still able to detect >90% true positive hits. We similarly used cancer dependency datasets to validate the ability of ZetaSuite in the absence of built-in controls. As for using prior expression profiles to obtain the list of non-expressors to calculate SS, it is easy to retrieve the data from many known databases, such as GEPIA(http://gepia.cancer-pku.cn/), Expression Atlas (https://www.ebi.ac.uk/gxa/home), Gene Expression Barcode database (<http://barcode.luhs.org>), etc. Moreover, we also provided non-expressor profiles in 38 cancer tissues (from TCGA), 54 normal tissues (from GTEx) as well as 1393 popular cancer/matched normal cell lines (from CCLE) in our website (https://github.com/YajingHao/ZetaSuite/tree/master/Non-expressors_profile). As for some new/rare cell types, users can either search the GEO database or directly generate RNA-seq data and then use classical RNA-seq processing pipelines provided in Homer(http://homer.ucsd.edu/homer/ngs/rnaseq/index.html) to generate the list of non-expressed genes.

In summary, although we developed ZetaSuite using our in-house dataset, we show that the Zeta statistics and associated ZetaSuite are broadly applicable to diverse types of two-dimensional high-throughput data. We also provided several ways to generate the list of non-expressors in our step-by-step user manual.

*5) A final critical omission from this manuscript is a clear guide for how a user can apply this method to their data. As a method, the manuscript does not include a guide to the data types and steps that it would take for readers to use or consider whether ZetaSuite is applicable to their data.*

In response to the suggestion of the Reviewer, we have fulfilled the gap by providing a step-by-step user manual in GitHub (https://github.com/YajingHao/ZetaSuite).

*Minor points:*

*1) The authors make frequent dispositive statements without providing proper citations, such as line 171 ("**well-known splicing regulator PTB"),*

Added.

*line 195 ("In certain applications, it may be necessary.." which applications?),*

I have modified the text to recommend the use of weighted Zeta scores in most applications.

*lines 221-22 ("FPL will gradually decrease while FNL will progressively increase.").*

Added.

*The last point in particular. The authors claim that* *with* *more readouts the noise would increase, that is counter-intuitive (it is not often that the noise of one data set resembles the noise of one generated by a different assay) the authors should demonstrate that is the case in more than one data set.*

The Reviewer might be confused with the situation where multiple biological and/or technical repeats under a given condition may help cancel experimental fluctuations.

This is not the case with our multi-target screening as each readout is independent of one another, thus each having the potential to produce a false positive result. Consequently, such noise will become accumulated with increasing number of readouts. We now used cancer dependency datasets to further illustrate our point. In both datasets, we first defined the cutoffs based on the scores of positive genes and then used the cutoffs to identify hits in these datasets. By plotting the specificity against the number of cell lines scored, we found that the specificity gradually decreased with the increasing number of cell lines scored (Fig.R5a-b). These results clearly illustrated accumulated noise with the increasing number of readouts scored.

Figure R5. Testing the specificity based on DepMap and DRIVE with different numbers of readouts.

*2) There are many grammatical errors, typos, and omissions throughout the manuscript which would benefit from careful editing. Examples include "Hit selection based on reflection points in the* *Screen Strength plot" (line 217), and "Any screen requires a cutoff to maximize true positives while minimize true negatives." (line 218), presumably you would want to maximize true negatives and minimize false negatives.*

Corrected.

**Reviewer #3:**

*1.* *Your paper does not provide proper account (either theoretical derivations or results from* *simulation studies) of the statistical operating characteristics of your proposed approach including a comparison to the* *statistical operating characteristics to other methods.* *How does a user assess the strength of the evidence in the results? You have a decision rule (e.g. the Strength Screen). What are its statistical properties (e.g., bias,* *error control)?*

In our manuscript, we introduced a new statistics, called Zeta, which is designed to treat two-dimensional data (i.e. many siRNA pools vs many functional readouts) by calculating the area under the screening results, in contrast to treatment of one-dimensional data (i.e. many siRNA pools vs a single functional readout) by using a single cut-off, like Z statistics and derivatives.

In the revised manuscript, we have further compared Zeta statistics with multiple other methods based on simulated datasets. As Zeta is designed to deal with random error accumulation due to increasing readout number, we generated a set of simulated data based on our two-dimensional splicing screening datasets by randomly selecting readouts from our raw datasets. We next evaluated the performance of the Zeta statistics in comparison with RSA, RIGER and MAGeCK, and now, additionally CB^2^ (see PMID: 31015259 and our response to Reviewer 2, main point 1) by calculating AUPRC values (Fig.R6). Note that AUPRC is more appropriate than AUROC when treating datasets where the number of negatives outweighs the number of positives. This analysis demonstrates that Zeta outperforms all existing methods, especially analyzing the data with large number of readouts, which has been incorporated as Fig.S3d in the revised manuscript.

Figure R6. Comparison among different methods in simulated datasets. Weighted ζ-scores in two directions calculated by ZetaSuite are combined in this analysis to reflect the overall functional consequence. As other software can only show changes in only one direction, we display the data separately for comparative purpose.

In our manuscript, we introduced the concept of Screen Strength (SS) to quantify the improvement in hit identification over random draw. As long as the selected threshold is picked by a positive SS, the errors obtained in the selected hits are decreased comparing to the ones in random draw. Higher SS value, lower errors obtained in the selected hits. If a screen is successful, more stringent cutoff would lead to a higher SS value but a lower total number of candidate hits. We then suggest using a balance point to make an optimal cutoff. The SS plot and the decision rule for hit selection depend on the data quality from a given experiment, which is estimated by the apparent FDR (not real FDR) and baseline FDR, as defined in our manuscript. In traditional hits selection, it uses the p-value from Z-score or derivations for testing no difference between a specific siRNA and negative controls to control errors (also called False Positive Level: the siRNAs with small effects to be selected as hits). As we proposed, in each genome-wide screening, we could generate a large number of internal negative controls (non-expressors). So, a given SS value can be converted to a specific FPL based on the pre-defined negative controls. But the same SS values likely represent different error rates in different experiments. Of note, the reason for not choosing FPL as the decision rule is that FPL is based on unknown numbers of true hits and true non-hits in a study. We found the errors in selected hits could still be very high even though the FPL is low (Fig.S4a). In contrast, SS is based on known numbers of selected hits and declared non-hits in a study, which provides more useful information. Therefore, the SS plot represents experiment-specific error rate to aid in hit selection, rather than a universal error model. Nevertheless, in our new SS plot, we add empirical FPL rules (FPL=0.01 and FPL= 0.05) and suggests that users could select the balance points above the specific empirical FPL rule according to their error tolerance.

*2. You state "**We herein introduce ZetaSuite, a statistical package initially designed to score hits from two-dimensional RNAi screens." This is very confusing. I assume that ZetaSuite is new package.*

Yes, ZetaSuite is a new package based on the newly developed Zeta statistics. We have now changed the statement to avoid confusion throughout the manuscript.

*3. Your github page hosts a large collection of mostly undocumented R and bash scripts. Given that you present this paper as software paper "ZetaSuite, a statistical package" you should consider producing a fully documented package. Looking at your wiki page, most of
the quantitative work was implemented in R so it would make sense to publish an R package to Bioconductor or CRAN. The example on the wiki page can serve as an initial version of the package* *vignette.*

Thanks for the suggestion. We have now packaged our method into a Perl module (https://github.com/YajingHao/ZetaSuite/ZetaSuite.pl) and submitted an R package under the name of ZetaSuite to CRAN (https://cran.r-project.org/web/packages/ZetaSuite/index.html).

*Moreover,* *you should provide a* *singularity or* *Docker definition file to reproduce the computational environment along with the scripts to reproduce every figure or table in your paper using this package.*

Our manuscript contains figures and tables generated by using ZetaSuite Perl module and R package as well as other figures and tables produced by other standard scripts. In this revised version, we now provide a singularity file to reproduce the computational environment along with the scripts to reproduce every figure or table. The file has been provided in Zenodo with DOI number: 10.5281/zenodo.6395174.

*4. Reading your paper, I was confused with your references to* *statistics, software package, methods and metrics.*

Our apology for the confusion. We have carefully checked the differences among statistics, software packages, methods, and metrics.

Statistics: Statistics is a process of designing, comparing, interpreting and analyzing data. It is often grouped into two broad categories – descriptive statistics and inferential statistics. The former refers to various techniques for summarizing and displaying data, such as mean and standard deviation. The latter includes procedures that make generalizations and draw conclusions based on the data’s information, such as Zeta statistics and Z statistics.

Software package: Package in which individual files or resources are packed together as a software collection that provides certain functionality, such as ZetaSuite Perl module or ZetaSuite R package.

Method: It literally means a prescribed process for completing a task, including a series of steps, or a collection of existing processes, taken to acquire knowledge, such as ZetaSuite.

Metrics: Metrics are quantitative measurements that focus on the quantitative meaningfulness of numbers, such as Zeta score and Screen Strength.

Based on these definitions, we have carefully proofread our manuscript to eliminate any potentially confusing statements.

*For example, L70: "Three methods were commonly used for such quality control (QC) purpose". I assume that you are referring to the three metrics (not methods) as you only list Seurat and Emptydrops (two). Also,* *Seurat is not a method. It is a software package.*

We mean to say “three QC methods implemented in Seurat, CellRanger, and EmptyDrops packages”.

*I assume that zeta "score" (L631) is the zeta statistic. But you also use the term "score" for the Z transformation*

Zeta score is Zeta statistic, which is the value calculated based on the Zeta statistics. Similarly, Z-score reflect the result of Z-transformation using the Z statistics. We have now corrected potential confusions in the revised manuscript.

*5. You state that L78: "In this study, we introduce* *a new zeta statistic by taking two critical QC metrics into consideration without relying on the selection of an arbitral cutoff." This gives the impression that there is an _existing_ class of statistics called zeta statistics and that you are introducing a new variant (this would be akin to saying we are developed a new score statistic or a new likelihood ratio statistic).*

Thanks for the suggestion. Corrected.

*But aside from that, I have concerns that you are not getting around arbitrary cutoffs. For example, you are using an arbitrary cutoff to declare an isoform detectable (L588: "in our study,* *a value is missing if one of the mRNA isoforms is undetectable").*

In our splicing screening, the ratio of two isoforms of the same mRNA was used as input, which requires the ability to detect both isoforms. To avoid potential confusion, we have modified our statement to “in our in-house dataset, the ratios are used as input and the ratio is missing if one of the mRNA isoforms is undetectable”.

*You are using a bin size of 100. Your method will likely be sensitive to the choices of these tuning parameters that you have picked in an arbitrary fashion without let's say cross-validation or using independent data sets.*

Bin size is used to calculate Zeta score with more bins to obtain a more accurate Zeta value as in Calculus (Fig.R7a). However, we don’t believe that the bin size dramatically affects the performance of the Zeta statistics in discriminating between hits and non-hits once the bin size reaches to certain resolution. To illustrate this point, we chose a range of different bin sizes (1,5,10,20,50,100) and checked the performance of the resulting Zeta scores. Using non-expressed genes as independent datasets, we found that the Zeta score calculated under different bin sizes showed similar PRC curves once bin size reaches 10 (Fig.R7b-c). In treating our datasets, we found that bin size=10 is sufficient. In order to calculate the area more accurately, we chose a larger bin size=100. Now, we have included this as a parameter in our ZetaSuite R package, and if needed, user has the freedom to choose a different bin size.

Figure R7. a. The diagram of zeta calculation with different bin sizes. b. and c. The PRC curves (b) and AUPRC values (c) with different bin sizes in HTS^2^ dataset.

*6. Why is the zeta score in L635* *subscripted by i (the left-hand side)?*

For each gene i, we calculate a Zeta score to reflect the regulatory strength based on the responses of all functional readouts.

*7. What is meant with |Z| < 2 implies P-value is > 0.05. What is Z? Is the Z score or Z statistics (again you use both the terms statistic L604 and score and L606) or does or denote a standard normal random variable? What is the sampling distribution? is* *|Z| < 2 a rejection region or is |Z|>2 a rejection region? This is another example where your* *statistical arguments are vague and hard to follow*

Z is the Z statistics in our manuscript except the “Z” in the formulas. The one in the formulas is the Z-score. We have proofread the manuscript to change Z to Z statistics. Z statistics is the traditional method for hits selection based on the mean absolute deviation in negative control samples. After Z-transformation according to the formula $(x-\mu)/\sigma$, the original values in a sample are transformed to Z-scores. Each Z-score tells us the exact location of the original value within the negative control sample distribution. According to the “Central limit theorem”: the sampling distribution tends to be normal if the sample is large enough (n>30), regardless of the shape of the data. Our negative controls have 510 samples. For each readout, the original values in negative control samples follow a normal distribution. We also randomly selected three readouts to draw Q-Q plots (Fig.R8). Because the original distribution is a normal one, the Z-transformed samples follow standard Gaussian distribution. Using |Z-score|=2 as a threshold and |Z-score|>2 as a rejection region, the corresponding P-value is 0.0455. The p-values are larger than 0.05 when choose |Z-score|<2 (for example |Z-score|=1.9 as a threshold, the P-value is 0.057) as thresholds. We hope the modified statistical arguments is sufficiently clear.

Figure R8. Q-Q plots of three randomly selected readouts.

1.	Tejedor JR, Papasaikas P, Valcarcel J: **Genome-wide identification of Fas/CD95 alternative splicing regulators reveals links with iron homeostasis.** *Mol Cell* 2015, **57:**23-38.

2.	Moore MJ, Wang Q, Kennedy CJ, Silver PA: **An alternative splicing network links cell-cycle control to apoptosis.** *Cell* 2010, **142:**625-636.

3.	McCormack NM, Abera MB, Arnold ES, Gibbs RM, Martin SE, Buehler E, Chen YC, Chen L, Fischbeck KH, Burnett BG: **A high-throughput genome-wide RNAi screen identifies modifiers of survival motor neuron protein.** *Cell Rep* 2021, **35:**109125.

4.	Chia NY, Chan YS, Feng B, Lu X, Orlov YL, Moreau D, Kumar P, Yang L, Jiang J, Lau MS, et al: **A genome-wide RNAi screen reveals determinants of human embryonic stem cell identity.** *Nature* 2010, **468:**316-320.

5.	Hasson SA, Kane LA, Yamano K, Huang CH, Sliter DA, Buehler E, Wang C, Heman-Ackah SM, Hessa T, Guha R, et al: **High-content genome-wide RNAi screens identify regulators of parkin upstream of mitophagy.** *Nature* 2013, **504:**291-295.

6.	Lun ATL, Riesenfeld S, Andrews T, Dao TP, Gomes T, participants in the 1st Human Cell Atlas J, Marioni JC: **EmptyDrops: distinguishing cells from empty droplets in droplet-based single-cell RNA sequencing data.** *Genome Biol* 2019, **20:**63.

7.	Ilicic T, Kim JK, Kolodziejczyk AA, Bagger FO, McCarthy DJ, Marioni JC, Teichmann SA: **Classification of low quality cells from single-cell RNA-seq data.** *Genome Biol* 2016, **17:**29.

8.	Luecken MD, Theis FJ: **Current best practices in single-cell RNA-seq analysis: a tutorial.** *Mol Syst Biol* 2019, **15:**e8746.

9.	Xi NM, Li JJ: **Benchmarking Computational Doublet-Detection Methods for Single-Cell RNA Sequencing Data.** *Cell Syst* 2021, **12:**176-194 e176.

10.	Sigoillot FD, Lyman S, Huckins JF, Adamson B, Chung E, Quattrochi B, King RW: **A bioinformatics method identifies prominent off-targeted transcripts in RNAi screens.** *Nat Methods* 2012, **9:**363-366.

**Second round of review**

**Reviewer 1**

The authors have addressed all my concerns.

**Reviewer 2**

The authors have adequately addressed my concerns in their revised manuscript and their thorough response to my original comments and concerns.
